# A Molecular Electron Density Theory Study of the Competitiveness of Polar Diels–Alder and Polar Alder-ene Reactions

**DOI:** 10.3390/molecules23081913

**Published:** 2018-07-31

**Authors:** Luis R. Domingo, Mar Ríos-Gutiérrez, Patricia Pérez

**Affiliations:** 1 Department of Organic Chemistry, University of Valencia, Moliner 50, 46100 Valencia, Spain; rios@utopia.uv.es; 2 Facultad de Ciencias Exactas, Departamento de Ciencias Químicas, Universidad Andres Bello, Av. República 498, Santiago 8370146, Chile; p.perez@unab.cl

**Keywords:** Molecular Electron Density Theory, Diels-Alder reactions, Alder-ene reactions, competitive reactions, polar reactions, Prins reaction, *pseudocyclic* selectivity

## Abstract

The competitiveness of the BF_3_ Lewis acid (LA) catalyzed polar Diels–Alder (P-DA) and polar Alder-ene (P-AE) reactions of 2-methyl-1,3-butadiene, a diene possessing an allylic hydrogen, with formaldehyde has been studied within the Molecular Electron Density Theory (MEDT) at the MPWB1K/6-311G(d,p) computational level. Coordination of BF_3_ LA to the oxygen of formaldehyde drastically accelerates both reactions given the high electrophilic character of the BF_3_:formaldehyde complex. As a consequence, these reactions present a very low activation enthalpy—less than 2.2 kcal·mol^−1^—thus becoming competitive. In dioxane, the P-AE reaction is slightly favored because of the larger polar character of the corresponding transition state structure (TS). In addition, the Prins reaction between hexahydrophenanthrene and the BF_3_:formaldehyde complex has also been studied as a computational model of an experimental P-AE reaction. For this LA-catalyzed reaction, the P-DA reaction presents very high activation energy because of the aromatic character of the dienic framework. The present MEDT study allows establishing the similarity of the TSs associated with the formation of the C–C single bond in both reactions, as well as the competitiveness between P-AE and P-DA reactions when the diene substrate possesses at least one allylic hydrogen, thus making it necessary to be considered by experimentalists in highly polar processes. In this work, the term “*pseudocyclic* selectivity” is suggested to connote the selective formation of structural isomers through stereoisomeric *pseudocyclic* TSs.

## 1. Introduction

**Scheme 1:**
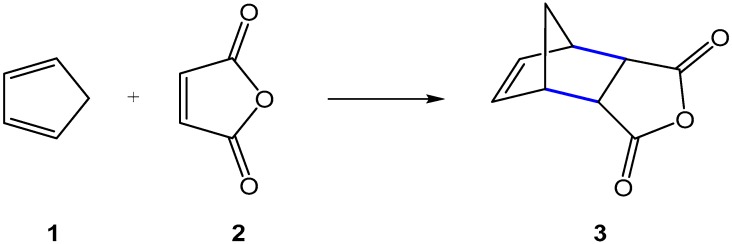
Diels-Alder reaction between cyclopentadiene **1** and maleic anhydride **2** yielding the bicyclic adduct **3** [1].

The Diels–Alder (DA) reaction between a conjugated diene and an ethylene yielding a cyclohexene derivative, reported for the first time by Otto Diels and Kurt Alder in 1927 [1], is one of the most studied organic reactions from a synthetic as well as theoretical point of view (see Scheme 1) [2,3].

**Scheme 2:**
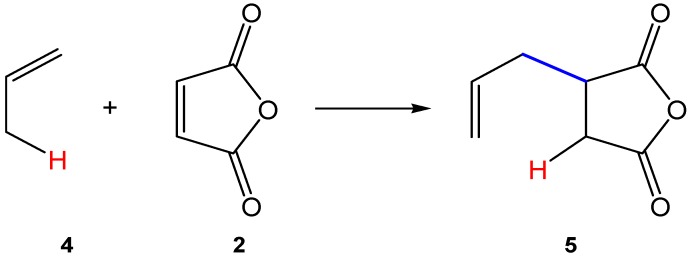
Alder-ene reaction of propene **4** with maleic anhydride **2** yielding adduct **5** [6].

On the other hand, the Alder-ene (AE) reaction, first reported by Kurt Alder in 1943 [4], which involves an alkene with an allylic C–H bond and an ethylene derivative, is one of the simplest ways to achieve the formation of a C–C single bond (see Scheme 2) [5].

Mechanistically, both DA and AE reactions were classified as pericyclic reactions [7,8], in which “all first order changes in bonding relationships take place in concert on a closed curve” [9]. However, a recent MEDT [10] study of the unfavorable DA and AE reactions of 1,3-butadiene **6** and propene **4** with ethylene **7** showed that the bonding changes in these reactions take place sequentially, and not in a simultaneous (concerted) cyclic rearrangement, thus ruling out the pericyclic mechanism [11]. That MEDT study emphasized that the high activation energies associated to these *pseudocyclic* reactions are due to the high-energy costs demanded for the rupture of the C–C double bonds present in the unsaturated hydrocarbon reagents.

**Scheme 3:**
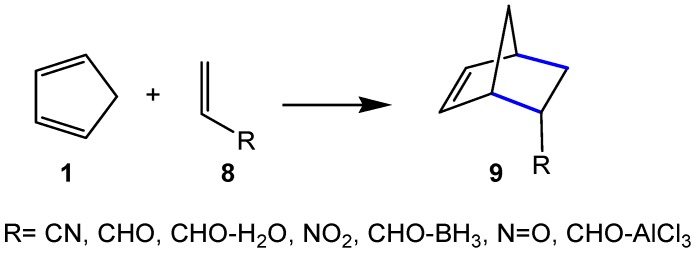
Series of polar Diels-Alder reactions between Cp **1** and ethylene derivatives **8** of increased electrophilicity.

A density functional theory [12] (DFT) study of the DA reactions between cyclopentadiene (Cp) **1** and a series of ethylenes **8** of increased electrophilic character, easily taking place experimentally, allowed establishing the polar mechanism in which the feasibility of DA reactions depends on their polar character; i.e., the increase of the nucleophilic character of the diene as well as the electrophilic character of the ethylene, or vice versa, making the reaction easier (see Scheme 3) [13]. Along this series of polar DA (P-DA) reactions, the increase of the polar character of the reaction, measured by the global electron density transfer [14] (GEDT) at the corresponding transition state structures (TSs), decreases the activation energies [15].

**Scheme 4:**
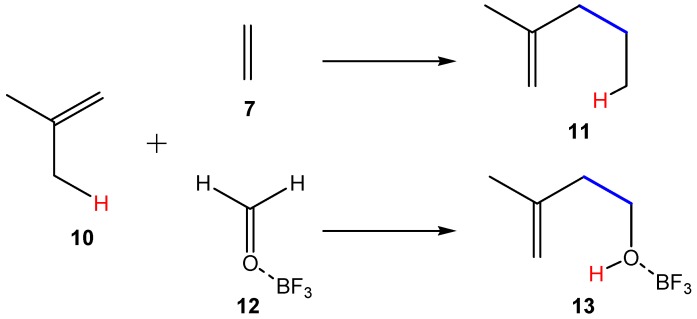
Alder-ene reactions of isobutene **10** with ethylene **7** or Lewis acids complex BF_3_:formaldehyde **12**.

The mechanism of the AE reactions between isobutene **10** and twelve ethylenes of increased electrophilicity was recently studied (see Scheme 4) [16]. Just as in P-DA reactions [13], a very good correlation between the activation energies and the GEDT computed at the corresponding TSs was found [16]; i.e., the more polar the AE reaction, the faster it is.

**Scheme 5:**
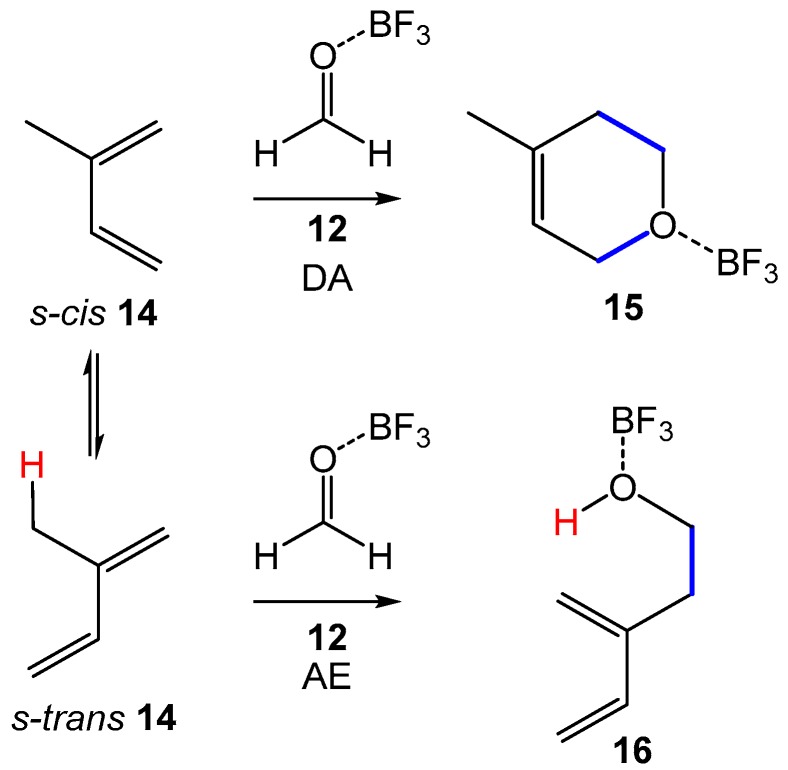
Competitive P-DA and P-AE reactions between the *s*-*trans* and *s*-*cis* conformations of 2-methyl-1,3-butadiene **14** and Lewis acid complex **12**, respectively.

The molecular mechanisms of the P-DA [13] and polar AE (P-AE) [16] reactions present a great similarity—both are initialized by the nucleophilic attack of the terminal carbon of the butadiene or the propene on the most electrophilic center of the ethylene derivative. The subsequent ring closure, in the case of the P-DA reaction, or hydrogen abstraction, in the case of the P-AE reaction, take place after passing the corresponding TS in a straightforward manner towards the final product of the reactions. Consequently, in molecules such as 2-methyl-1,3-butadiene (2MBD) **14**, which possesses a 1,3-butadiene framework and at least one allylic hydrogen, both P-DA and P-AE reactions can be competitive pathways (see Scheme 5).

**Scheme 6:**
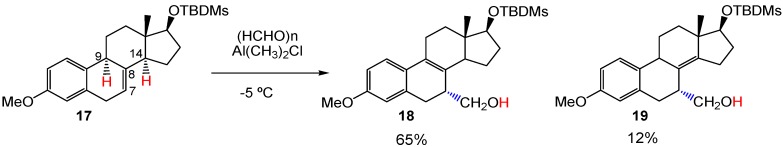
Prins reaction of the steroidal olefin **17** [18].

In this sense, in 1991 Künzer et al. studied the dimethylchloride aluminium LA-mediated Prins reaction [17] of steroidal olefin **17** with paraformaldehyde yielding homoallylic alcohols **18** and **19** (see Scheme 6) [18]. The DA cycloadduct involving the aromatic ring of steroidal olefin **17** was not observed.

This LA-mediated Prins reaction can mechanistically be considered a P-AE reaction involving the C7–C8 double bond and the allylic H9 and H14 hydrogens of steroidal olefin **17**, and the LA complex formed between Al(CH_3_)_2_Cl and formaldehyde **20**. In this P-AE reaction, the hydrogen transfer process from the C9 and C14 carbons of steroidal olefin **17** to the carbonyl oxygen of the corresponding LA complex yields a homoallylic alcohol.

**Scheme 7:**
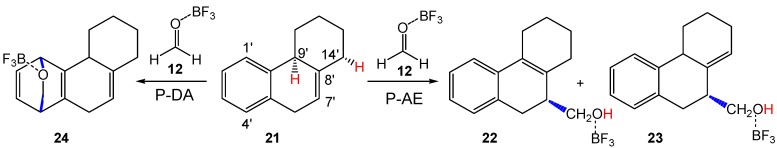
Competitive polar Diels-Alder and polar Alder-ene reactions between hexahydrophenanthrene **21** and Lewis acid complex BF_3_:formaldehyde **12**.

In order to investigate the possible competition between P-DA and P-AE reactions, the reaction between 2MBD **14** and LA complex BF_3_:formaldehyde **12** yielding the P-DA cycloadduct **15** and/or the P-AE adduct **16** are first studied within MEDT at the MPWB1K/6-311G(d,p) computational level (see Scheme 5). Then, the P-AE reaction of hexahydrophenanthrene (HPA) **21** with LA complex **12**, as a reduced model of the Prins reaction experimentally carried out by Künzer et al. [18], as well as the P-DA reaction involving the aromatic ring of **21**, are also studied (see Scheme 7).

## 2. Computational Methods

A recent analysis of the applicability of the B3LYP [19,20], MPWB1K [21], and M06-2X [22] functionals in the study of non-polar and polar cycloaddition reactions allowed selecting the MPWB1K functional as the most adequate one for the study of this type of organic reactions [15]. Consequently, DFT calculations were performed by using the MPWB1K functional together with the 6-311G(d,p) basis set [23]. Optimizations were carried out using the Berny analytical gradient optimization method [24,25]. The stationary points were characterized by frequency computations in order to verify that TSs have one and only one imaginary frequency. The intrinsic reaction coordinate (IRC) paths [26] were traced in order to check and obtain the energy profiles connecting each TS to the two associated minima of the proposed mechanism using the second order González-Schlegel integration method [27,28]. Solvent effects of 1,4-dioxane in the competitive P-DA and P-AE reactions of 2MBD **14** with LA complex BF_3_:formaldehyde **12** were taken into account by full optimization of the gas phase structures at the MPWB1K/6-311G(d,p) computational level using the polarizable continuum model (PCM) developed by Tomasi’s group [29,30] in the framework of the self-consistent reaction field (SCRF) [31,32,33]. As solvent effects do not produce remarkable changes neither in energies nor in geometries, solvent effects in the P-AE and P-DA reactions between HPA **21** and LA complex **12** were taken into account by single point energy calculations using the gas phase structures.

The GEDT was computed by the sum of the natural atomic charges (q), obtained by a natural population analysis [34,35] (NPA), of the atoms belonging to each framework (f) at the TSs; i.e., GEDT (f) = $\sum_{q\in f}q$; f = nucleophile, electrophile. The sign indicates the direction of the electron density flux in such a manner that positive values mean a flux from the considered framework to the other one. Conceptual DFT (CDFT) global reactivity indices [36,37] and Parr functions [38] were computed using the equations given in reference 37. All computations were carried out with the Gaussian 09 suite of programs [39].

Topological analysis of the electron localization function [40] (ELF) was performed with the TopMod [41] package using the corresponding MPWB1K/6-311G(d,p) monodeterminantal wave functions and considering a cubical grid of step size of 0.1 Bohr, while quantum theory of atoms in molecules [42] (QTAIM) studies were performed with the Multiwfn [43] program. The molecular geometries and ELF basin attractor positions were visualized using the GaussView program [44], while the representation of the ELF basin isosurfaces was done by using the UCSF Chimera program [45].

## 3. Results and Discussion

The present MEDT study has been divided into four sections: in Section 3.1, an analysis of the CDFT reactivity indices at the ground state (GS) of the reagents involved in the competitive P-DA and P-AE reactions given in Scheme 5 and Scheme 7 is carried out; in Section 3.2, the competitive P-DA and P-AE reactions between 2MBD **14** and LA complex **12** are studied; in Section 3.3, the reaction paths associated to the P-DA and P-AE reactions between HPA **21** and LA complex **12** are explored and characterized; and finally, in Section 3.4, a comparative ELF topological analysis of the stationary points involved in the competitive P-DA and P-AE reactions between 2MBD **14** and LA complex **12** is performed.

### 3.1. Analysis of the CDFT Reactivity Indices at the GS of the Reagents

Numerous studies devoted to DA and AE reactions have shown that the analysis of the reactivity indices defined within CDFT [36,37] is a powerful tool to predict the reactivity in polar reactions. Consequently, in order to characterize the reactivity of *s*-*cis* and *s*-*trans* 2MBDs **14**, HPA **21**, formaldehyde **20**, and LA complex **12** in polar reactions, the CDFT reactivity indices at the GS of the reagents were analysed. The global indices, namely, the electronic chemical potential, μ, chemical hardness, η, electrophilicity, ω, and nucleophilicity, *N*, are given in Table 1.

The electronic chemical potentials [46,47] μ of *s*-*cis* and *s*-*trans* 2MBDs **14** and HPA **21**, μ = −3.30 (*s*-*cis* **14**), −3.31 (*s*-*trans* **14**) and −2.95 (**21**) eV, are significantly higher than that of LA complex **12**, μ = −5.93 eV, indicating that along a polar reaction, the GEDT [14] will flux from these ethylene derivatives towards the electrophilic LA complex **12**.

The electrophilicity ω [48] and nucleophilicity *N* [49] indices of formaldehyde **20** are ω = 1.45 and *N* = 1.81 eV, thus being classified as a strong electrophile within the electrophilicity scale [50] and as a moderate nucleophile within the nucleophilicity scale [51]. Note that within the group of strong electrophiles, there are experimentally stronger and weaker electrophiles. Thus, although formaldehyde **20** is classified as a strong electrophile within the electrophilicity scale, its electrophilic character is not strong enough to favor a polar reaction experimentally. Coordination of LA BF_3_ to the carbonyl oxygen of formaldehyde **20** notably increases the electrophilicity of the LA complex **12** to 2.69 eV. This strong electrophilic activation accounts for the participation of this carbonyl compound in polar reactions. On the other hand, the electrophilicity ω indices of the unsaturated hydrocarbons are 0.94 (*s*-*cis* **14**), 0.99 (*s*-*trans* **14**) and 0.71 (**21**) eV, 2MBD **14** being classified as a moderate electrophile and HPA **21** as a marginal electrophile, while their nucleophilicity *N* indices, 2.94 (*s*-*cis* **14**), 3.07 (*s*-*trans* **14**) and 3.11 (**21**) eV, allow the classification of *s*-*cis* 2MBD **14** on the borderline of strong nucleophiles, and *s*-*trans* 2MBD **14** and HPA **21** as strong nucleophiles. Consequently, along a polar reaction, it is expected that LA complex **12** will act as a strong electrophile, while these unsaturated hydrocarbons will act as strong nucleophiles.

**Figure 1:**
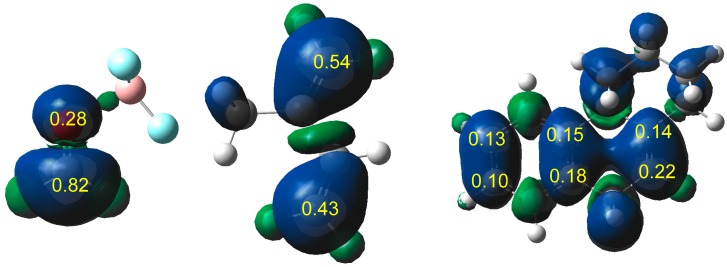
Three-dimensional representations of the Mulliken atomic spin densities of radical anion **12**^−^ and radical cations *s*-*trans* 2MBD **14**^+^ and HPA **21^+^**, together with the electrophilic $P_{k}^{+}$ and nucleophilic $P_{k}^{-}$ Parr functions of LA complex **12** and unsaturated hydrocarbons *s*-*trans* 2MBD **14** and HPA **21**.

By approaching nonsymmetric electrophilic/nucleophilic pairs along a polar process, the most favorable reactive pathway is that associated with the initial two-center interaction between the most electrophilic center of the electrophile and the most nucleophilic center of the nucleophile. Recently, Domingo et al. proposed the electrophilic $P_{k}^{+}$ and nucleophilic $P_{k}^{-}$ Parr functions [38], derived from the changes of spin electron density reached via the GEDT process from the nucleophile to the electrophile, as a powerful tool to study the local reactivity in polar and ionic processes. Accordingly, the electrophilic $P_{k}^{+}$ Parr functions of LA complex **12**, as well as the nucleophilic $P_{k}^{-}$ Parr functions of unsaturated hydrocarbons *s*-*trans* 2MBD **14** and HPA **21**, were analyzed in order to characterize the most electrophilic and nucleophilic centers of the species involved in the addition reaction of LA complex **12** to unsaturated hydrocarbons *s*-*trans* 2MBD **14** and HPA **21** (see Figure 1).

**Scheme 8:**
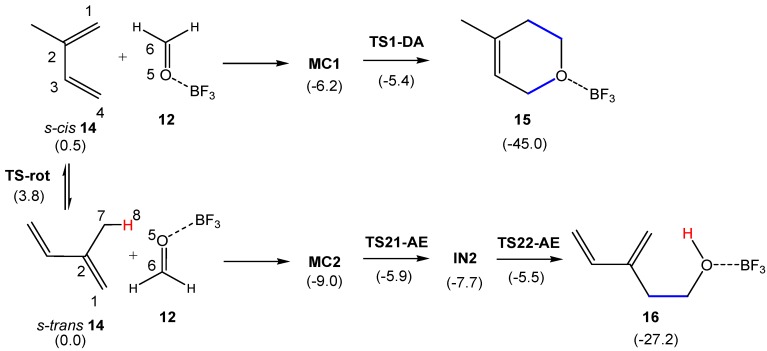
Competitive polar Diels-Alder and polar Alder-ene reaction paths associated to the reaction of 2-methyl-1,3-butadiene **14** and Lewis acid complex **12** along the more favorable C1–C6 regioisomeric pathways. Relative energies in dioxane, in kcal·mol^−1^, are given in parentheses.

Analysis of the electrophilic $P_{k}^{+}$ Parr functions at the reactive sites of LA complex **12** indicates that the carbonyl carbon, with a $P_{k}^{+}$ value of 0.82, is the most electrophilic center of this species. On the other hand, analysis of the nucleophilic $P_{k}^{-}$ Parr functions at the reactive sites of *s*-*trans* 2MBD **14** indicates that the C1 carbon, $P_{k}^{-}$ = 0.54, is more nucleophilically activated than the C4 carbon, $P_{k}^{-}$ = 0.43, although slightly (see Scheme 8 for nuclei labels). Finally, analysis of the nucleophilic $P_{k}^{-}$ Parr functions at the reactive sites of HPA **21** shows at least six nucleophilically activated centers, the C7′ carbon, $P_{k}^{-}$ = 0.22, being the most nucleophilic one. Consequently, it is expected that the P-AE reaction begins with the electrophilic attack of the carbonyl carbon of LA complex **12** on the C4′ or C7′ carbons of unsaturated hydrocarbons *s*-*trans* 2MBD **14** or HPA **21**, respectively, in clear agreement with the experimental outcomes (see Scheme 6) [18].

### 3.2. Comparative Study of the Competitive P-DA and P-AE Reactions between 2MBD ***14*** and LA Complex ***12***

In this section, the competitive P-DA and P-AE reaction paths associated with the BF_3_ LA-catalyzed reactions between 2MBD **14** and formaldehyde **20** are studied. For comparative purposes, the non-catalyzed DA and AE reactions between 2MBD **14** and formaldehyde **20** were also studied; the corresponding results are given in Supplementary Information. Analysis of the stationary points involved in the more favorable C1–C6 regioisomeric reaction paths associated with the two competitive P-DA and P-AE reaction paths shows that while the P-DA reaction path is characterized by a two-step mechanism similar to that found in the non-catalyzed reaction, the P-AE reaction path is characterized by a three-step mechanism (see Scheme 8). MPWB1K/6-311G(d,p) total and relative electronic energies, in gas phase and in dioxane, of the stationary points involved in the two competitive reaction paths are given in Table S4 in Supplementary Information. Relative energies in dioxane are given in Scheme 8.

Similar to the non-catalyzed process, the first step of both reaction paths is the formation of a molecular complex (MC) in which LA complex **12** is located above the plain of *s*-*cis* 2MBD **14** at a distance of ca. 2.6 Å. These species are found 6.2 (**MC1**) and 9.0 (**MC2**) kcal·mol^−1^ below the reagents. Interestingly, these MCs are more stable than those found in the non-catalyzed process presenting an O5–H8′ hydrogen bond (see Supplementary Information). From **MC1**, formation of cycloadduct **15** takes place along one elementary step via **TS1-DA**. The activation energy associated with **TS1-DA** from the more stable **MC2** is only 3.6 kcal·mol^−1^; from **MC2**, formation of the final cycloadduct **15** being strongly exothermic by 36.0 kcal·mol^−1^.

Along the P-AE reaction path, the mechanism experiences a significant change: after the electrophilic attack of LA complex **12** on *s*-*trans* 2MBD **14**, an intermediate **IN2** associated with the formation of the C1–C6 single bond is found, thus making the formation of **16** from **MC2** a stepwise process. From the more stable **MC2**, the activation energy associated to **TS21-AE** is 3.1 kcal·mol^−1^, formation of intermediate **IN2** being endothermic by 1.3 kcal·mol^−1^. From **IN2**, the hydrogen transfer process via **TS22-AE** presents an activation energy of only 2.2 kcal·mol^−1^. From **MC2**, formation of the final adduct **16** is exothermic by 18.2 kcal·mol^−1^. 

A comparative analysis of the activation energies associated with the non-catalyzed and catalyzed reactions allows obtaining two appealing conclusions: (i) the presence of the BF_3_ LA catalyst produces a large acceleration of both P-DA and P-AE reactions as a consequence of the strong electrophilic character of LA complex **12** (see Table 1) [13,16]. In fact, all the stationary points associated with the two catalyzed reaction paths are energetically found below the separated reagents; (ii) in the catalyzed process, **TS22-AE** is located 0.1 kcal·mol^−1^ below **TS1-DA**. Consequently, in dioxane, both P-DA and P-AE reactions become competitive in the catalyzed process.

**Figure 2:**
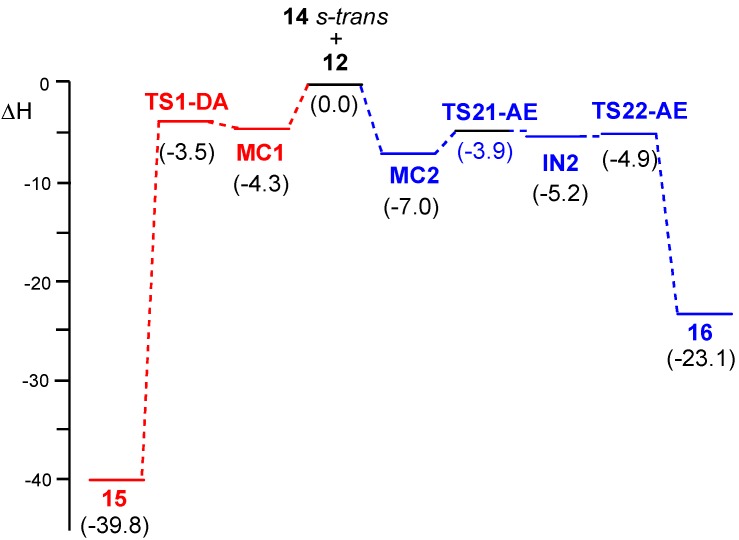
MPWB1K/6-311G(d,p) enthalpy profiles, kcal·mol^−1^, computed at −5 °C in dioxane, of the competitive P-DA and P-AE reaction paths associated with the reaction of 2MBD **14** with LA complex **12** along the more favorable C1–C6 regioisomeric pathways.

In order to investigate how thermal corrections can modify the relative electronic energies, thermodynamic calculations were performed for the two competitive P-DA and P-AE reaction paths. Thermodynamic data are given in Table S5 of Supplementary Information, while the enthalpy profiles of the two competitive P-DA and P-AE reaction paths are represented in Figure 2. As this figure shows, **MC1** and **MC2** are minima on the enthalpy profile. On the other hand, analysis of the P-AE enthalpy profile emphasizes that **TS21-AE**, **IN2** and **TS22-AE** present similar relative enthalpies. Interestingly, when the thermal corrections are considered, **TS21-AE**, which determines the rate-determining step (RDS) of the P-AE reaction path, is found 0.4 kcal·mol^−1^ below **TS1-DA**. This behavior indicates that, under kinetic control, adduct **16** resulting from the P-AE reaction will be formed to a slightly larger extent than cycloadduct **15** resulting from the P-DA reaction. The latter would be the product of a thermodynamic control of the reaction between 2MBD **14** and LA complex **12**.

**Figure 3:**
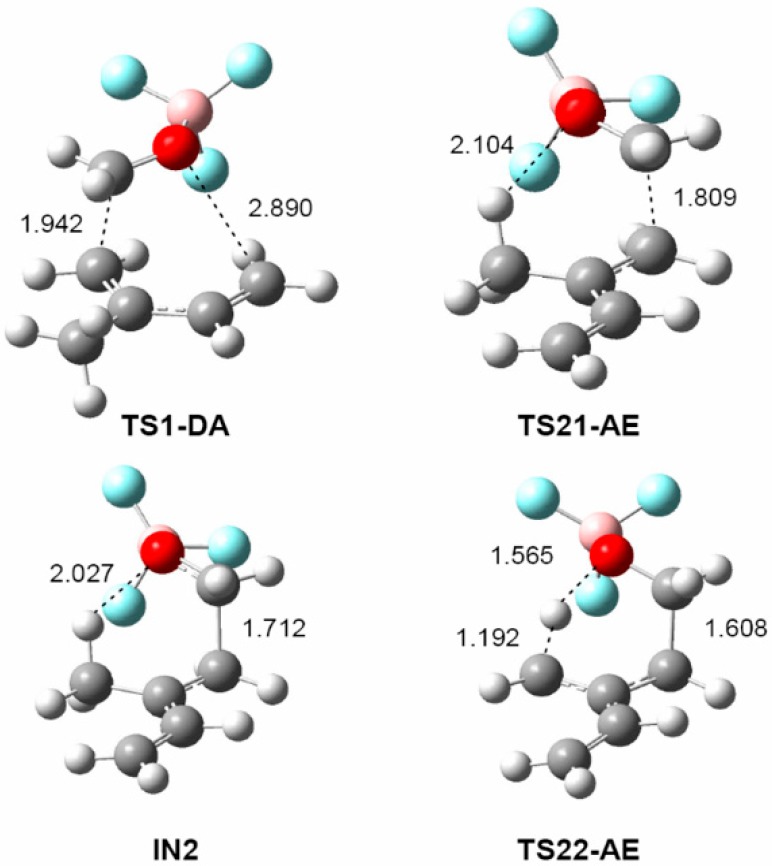
MPWB1K/6-311G(d,p) gas phase optimized geometries of the TSs and intermediate involved in the more favorable C1–C6 regioisomeric pathways associated to the competitive P-DA and P-AE reactions between 2MBD **14** and LA complex **12**. Distances are given in angstroms, Å.

The gas phase geometries of the TSs involved in the competitive P-DA and P-AE reactions between 2MBD **14** and LA complex **12** are displayed in Figure 3. At **TS1-DA**, the large difference between the C–C and C–O distances, 0.95 Å, suggests that this TS may be related to a *two*-*stage one*-*step* mechanism [52], in which **TS1-DA** is associated to the electrophilic attack of the carbonyl C6 carbon of LA complex **12** on the C1 carbon of *s*-*cis* 2MBD **14**. At **TS21-AE**, the short C1–C6 distance, 1.809 Å, indicates that at this TS the C1–C6 single bond may be already formed [14]. The C1–C6 and O5–H8 distances at intermediate **IN2**, are slightly shorter than those at **TS21-AE**. The geometrical similarity between **TS21-AE** and **IN2** accounts for the notable flat PES found around these two species.

Finally, the electronic nature, i.e., polar character, of the competitive P-DA and P-AE reactions between 2MBD **14** and LA complex **12** was analyzed by computing the GEDT at the TSs and the intermediate. The values of the GEDT, fluxing from the butadiene to the LA:formaldehyde frameworks, are: 0.40 e at **TS1-DA**, 0.49 e at **TS21-AE**, 0.57 e at **IN2**. These high values allow establishing the high polar character of these reactions, which is a consequence of the strong nucleophilic character of 2MBD **14** and the strong electrophilic character of LA complex **12**, and which accounts for the low computed activation energies [15]. Again, the higher GEDT found at **TS21-AE** than at **TS1-DA** is a consequence of the more advanced character of the former. The considerably stronger polar character of the reaction involving LA complex **12** than that involving formaldehyde **20** (see Supplementary Information) accounts for the lower activation energies of the former.

### 3.3. Study of the P-AE and P-DA Reactions between HPA ***21*** and LA Complex ***12***

**Scheme 9:**
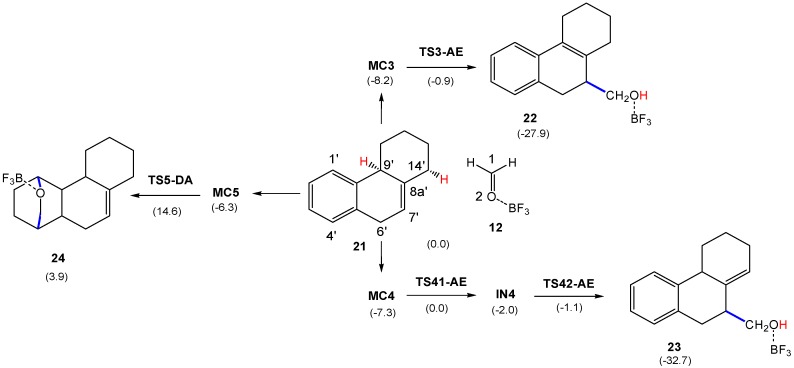
Competitive polar Alder-ene reactions between hexahydrophenanthrene **21** and Lewis acid complex **12** along the more favorable C1–C7′ regioisomeric pathway. The experimentally not observed polar Diels-Alder reaction involving the aromatic ring is also included. Relative energies in dioxane, in kcal·mol^−1^, are given in parentheses.

Next, the reaction between HPA **21** and LA complex **12**, as reduced compound models of the steroidal olefin **17** and the LA complex Al(CH_3_)_2_Cl:**20** experimentally used by Künzer (see Scheme 6) [18], was studied. As HPA **21** presents three allylic hydrogens at the 6′, 9′ and 14′ positions, three different P-AE reaction paths are feasible. However, according to the analysis of the Parr functions (see Section 3.1), only the more favorable P-AE reaction paths involving the most nucleophilic C7′ carbon were studied. In addition, the P-DA reaction involving the aromatic ring of HPA **21** was also considered. Analysis of the stationary points involved in the more favorable C1–C7′ regioisomeric pathway associated to the two competitive P-AE reactions involving the H9′ or H14′ hydrogens indicates that they have different mechanisms. While the reaction path involving the abstraction of the tertiary 9′ hydrogen takes place through a two-step mechanism, that involving the abstraction of the secondary 14′ hydrogen takes place through a three-step mechanism (see Scheme 9). On the other hand, the P-DA reaction involving the aromatic ring takes place through a two-step mechanism. MPWB1K/6-311G(d,p) total and relative electronic energies, in gas phase and in dioxane, of the stationary points involved in the three reaction paths are given in Table S6 in Supplementary Information. Relative energies in dioxane are given in Scheme 9.

The two competitive P-AE reaction paths begin with the formation of two MCs found 8.2 (**MC3**) and 7.3 (**MC4**) kcal·mol^−1^ below the separated reagents, which are found in thermodynamic equilibrium. The activation energy associated with the P-AE reaction path via **TS3-AE** is 7.3 kcal·mol^−1^; formation of the corresponding homoallylic alcohol **22** being exothermic by 19.7 kcal·mol^−1^. A more complex mechanism is found for the formation of homoallylic alcohol **23**; from **MC4**, the P-AE reaction path takes place via a two-step mechanism. After formation of **MC4**, the P-AE reaction begins by the electrophilic attack of the carbonyl carbon of LA complex **12** on the C7′ carbon of HPA **21**, yielding intermediate **IN4** via **TS41-AE**. From the most stable **MC3**, the activation energy associated to this step is 8.2 kcal·mol^−1^, formation of intermediate **IN4** being endothermic by 6.2 kcal·mol^−1^. The last step is the hydrogen transfer from the C14′ carbon of the HPA framework to the carbonyl oxygen of the formaldehyde one, yielding the final homoallylic alcohol **23**. The activation energy associated to this hydrogen transfer step is 0.9 kcal·mol^−1^, formation of the final homoallylic alcohol **23** being exothermic by 25.4 kcal·mol^−1^.

The P-DA reaction path begins with the formation of **MC5**, which is found 6.3 kcal·mol^−1^ below the separated reagents. From this MC, formation of cycloadduct **24** takes place in one elementary step via **TS5-DA**. From the most stable **MC3**, the activation energy associated to this P-DA reaction is 22.8 kcal·mol^−1^, the reaction being endothermic by 12.1 kcal·mol^−1^.

Some appealing conclusions can be drawn from the energy results given in Scheme 9: (i) these LA-catalyzed Prins (AE) reactions present very low activation energies, less than 8.2 kcal·mol^−1^, in agreement with the low temperature used in the reaction, −5 °C [18]; (ii) in dioxane, **TS3-AE** is slightly more stabilized than **TS41-AE** and, consequently, formation of adduct **22** is slightly more favorable than formation of **23**, in agreement with the experimental results (see Scheme 6); and finally, (iii) the P-DA reaction involving the participation of the aromatic ring presents a very high activation energy, similar to that found in the non-catalyzed process, making this P-DA reaction non-competitive with the P-AE reactions. This behavior is a consequence of the difficulty of the aromatic ring to participate as diene or ethylene in P-DA reactions [53]. Note that the activation energy associated to this P-DA reaction is 17.0 kcal·mol^−1^ higher than that involving 2MBD **14**.

**Figure 4:**
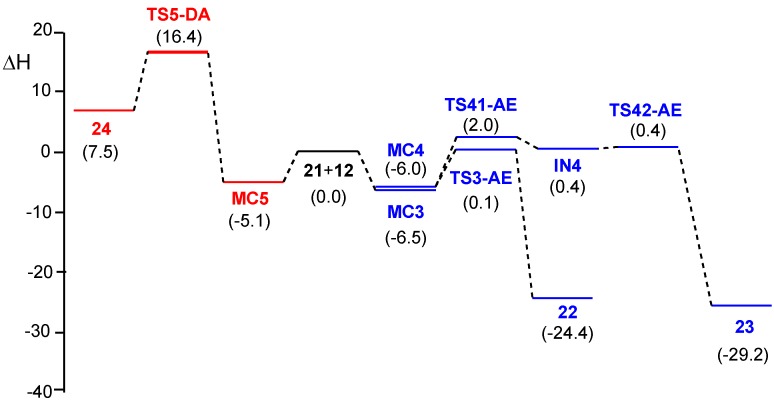
MPWB1K/6-311G(d,p) enthalpy profiles, kcal·mol^−1^, computed at −5 °C in dioxane, of the competitive P-DA and P-AE reaction paths associated with the reaction of between HPA **21** and LA complex **12**.

Thermodynamic data for the competitive reaction paths between HPA **21** and LA complex **12** are given in Table S7 of Supplementary Information, while the enthalpy profiles of the three competitive reaction paths are represented in Figure 4. As can be seen, the three MCs are minima on the enthalpy profile. The enthalpy profiles associated to the two competitive P-AE reactions show that **TS3-AE** is located 1.9 kcal·mol^−1^ below **TS41-AE**, in agreement with the preferential formation of homoallylic alcohols **18** (see Scheme 6) [18]. Note that the first step is the RDS of the stepwise AE reaction. On the other hand, the high activation enthalpy associated with **TS5-DA** makes this reaction path non-competitive. Formation of homoallylic alcohols **22** and **23** is strongly exothermic and, consequently, irreversible, while formation of the DA cycloadduct **24** is endothermic and, therefore, reversible. Thus, the DA reaction is both kinetically and thermodynamically unfavorable. Finally, a comparative analysis of the enthalpy profiles associated to the competitive P-AE reactions given in Figure 2 and Figure 4 shows a great similitude, supporting the reaction between 2MBD **14** and LA complex **12** as a good computational model of the reaction between HPA **21** and LA complex **12**.

**Figure 5:**
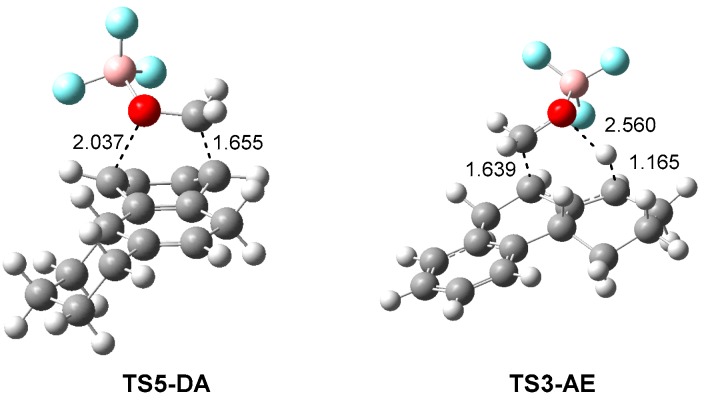

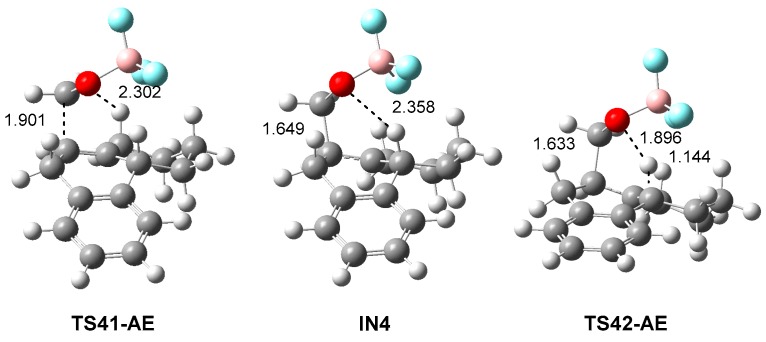
MPWB1K/6-311G(d,p) gas phase optimized geometries of the TSs and intermediate involved in the P-AE and P-DA reactions between HPA **21** and LA complex **12**. Distances are given in angstroms, Å.

The gas phase geometries of the TSs involved in the P-DA and P-AE reactions between HPA **21** and LA complex **12** are displayed in Figure 5. At **TS3-AE**, the lengths of the C1–C7′, C9′–H and O2–H indicate that this TS is mainly associated to the hydrogen transfer process; note that at intermediate **IN4**, the length of the C1–C7′ bond indicates that it is already formed [14]. At the stepwise P-AE reaction path, the C1–C7′ and O2–H distances at **TS41-AE**, 1.901 and 2.302 Å, respectively, indicate that this TS is associated to the formation of the C–C single bond. At intermediate **IN4**, the C1–C7′ bond length, 1.649 Å, indicates that this C–C single bond has practicality been formed. Finally, at **TS42-AE,** the C14′–H and O2–H lengths indicate that, similar to **TS42-AE**, this TS is mainly associated to the rupture of the C14′–H bond. Finally, at **TS5-DA**, the C1–C4′ and O2–C1′ lengths, 1.655 and 2.037 Å, respectively, reveal the very advanced character of this unfavorable TS, in which the C1–C4′ single bond has already been formed.

Finally, the electronic nature of the P-AE and P-DA reactions between HPA **21** and LA complex **12** was analyzed by computing the GEDT at the TSs and the intermediate. The values of the GEDT, fluxing from the HPA to the BF_3_:formaldehyde frameworks, along the P-AE reactions are 0.65 e (**TS3-AE**), 0.44 e (**TS41-AE**) and 0.64 e (**IN4**). These very high values allow establishing the strong polar character of these P-AE reactions, which accounts for the low computed activation energies. On the other hand, along the P-DA reaction, the value of the GEDT at **TS5-DA** is 0.52 e. Note that despite this very high GEDT value, the unfavorable activation energy associated to this TS is the consequence of the loss of the phenyl aromaticity [53]. The high polar character of these P-DA and P-AE reactions is the consequence of the strong nucleophilic character of HPA **21** and the strong electrophilic character of LA complex **12**.

### 3.4. Comparative ELF Analysis of the Competitive Polar P-DA and P-AE Reactions between 2MBD ***14*** and LA Complex ***12***. Origin of the Pseudocyclic Selectivity

In order to compare the C–C bond-formation process along the competitive P-DA and P-AE reactions between 2MBD **14** and LA complex **12**, a topological analysis of the ELF of the stationary points involved in the two reaction paths was carried out. The complete ELF analysis is given in Section 2 of the Supplementary Material.

**Figure 6:**
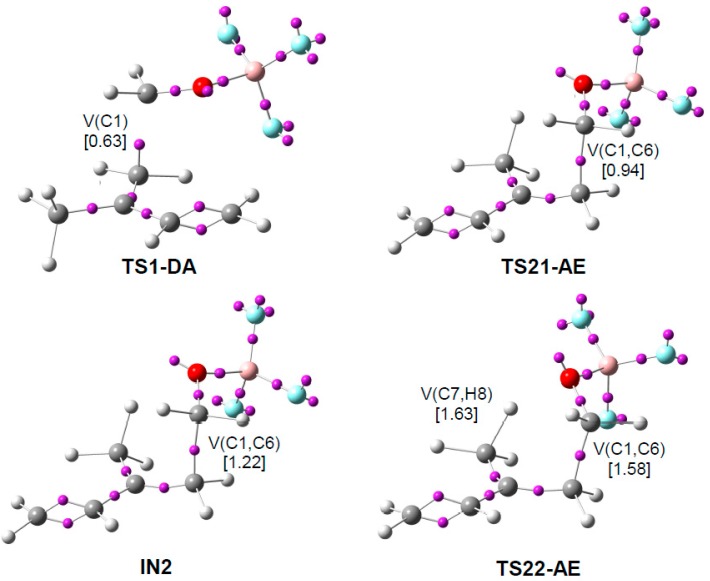
Attractor positions of the ELF valence basins for the TS involved in the P-DA reaction, **TS1-DA**, and TSs and intermediate involved in the P-AE reaction, **TS21-AE**, **IN2** and **TS22-AE**, between 2MBD **14** and LA complex **12**. The electron populations, in average number of electrons, e, are given in brackets.

Some appealing conclusions can be drawn from this comparative ELF analysis: (i) **TS1-DA** and **TS21-EA** present a great electronic similarity. The only topological difference between these TSs is the presence of the V(C1) monosynaptic basin with a population of 0.63 e at **TS1-DA** and the presence of the V(C1,C6) disynaptic basin with a population of 0.94 e at **TS21-AE**. Note that the V(C1) monosynaptic basin is demanded for the subsequent formation of the V(C1,C6) disynaptic basin. This topological difference, which was supported by a QTAIM analysis of the two TSs (see Supplementary Information), is a consequence of the more advanced character of the latter TS; (ii) consequently, **TS1-DA** and **TS21-EA** are a pair of stereoisomeric TSs with a very similar electronic structure (see Figure 6). This behavior accounts for their similar electronic energies; (iii) at **IN2**, while the new C1–C6 single bond has reached a population of 1.22 e, the population of the C7–H8 bond remains 1.86 e. Consequently, the first step of the P-AE reaction path is associated only to the formation of the C1–C6 single bond, in agreement with the geometrical analysis; and finally (iv) at **TS22-AE**, while the new C1–C6 single bond continues increasing its population to 1.58 e, the population of the C7–H8 bond still is 1.63 e, indicating that the rupture of the C7–H8 bond has not yet begun at this TS.

**Figure 7:**
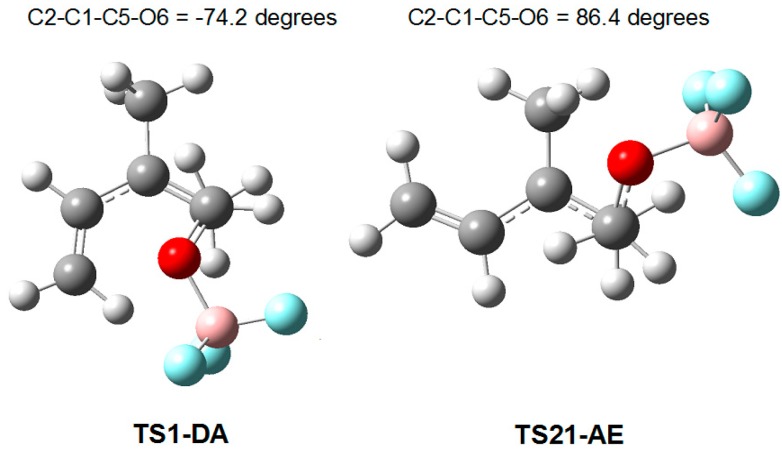
A view of **TS1-DA** and **TS21-AE** along the formation of the first C1–C5 single bonds. They are a pair of stereoisomeric TSs resulting from the C2–C3 and C1–C5 bond rotations.

A representation of the geometries of the competitive **TS1-DA** and **TS21-AE** along the formation of the first C1–C5 single bond shows that they are a pair of conformational (stereoisomeric) TSs resulting from the C2–C3 and C1–C5 single bond rotation, with a very similar electronic structure (see Figure 7). This behavior makes it possible to explain their similar electronic energy, and consequently, the competitiveness found between the DA and AE reaction paths.

As the two conformational TSs are associated with the nucleophilic attack of the C1 carbon of 2MBD **14** on the carbonyl C6 carbon of LA complex **12**, the two competitive reaction paths are differentiated after passing these TSs; while the P-DA reaction path yields cycloadduct **15** through the subsequent ring closure, the P-AE reaction path stops at intermediate **IN2**, which is able to take away a hydrogen of the neighboring methyl group with a very low activation energy yielding the final adduct **16**.

**Scheme 10:**
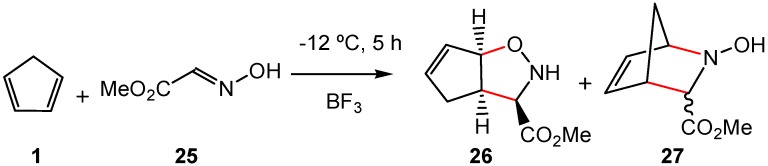
Lewis acid catalyzed reaction between cyclopentadiene **1** and methyl glyoxylate oxime **25** yielding the [3 + 2] or [2 + 4] cycloadducts **26** and **27**.

A similar finding was found in the competitive formation of the [3 + 2] or [2 + 4] cycloadducts **26** and **27** in the BF_3_ LA-catalyzed reaction between Cp **1** and methyl glyoxylate oxime **25** (see Scheme 10) [54]. This reaction was characterized by the nucleophilic attack of Cp **1** onto the carbon of the corresponding BF_3_:nitrone complex. After passing the competitive stereoisomeric TSs, the subsequent ring closure at the end of the reaction allows the formation of the [3 + 2] or [2 + 4] cycloadducts **26** and **27** [55]. These competitive cycloaddition reactions, which take place through non-concerted cyclic TSs, are classified as *pseudocyclic* reactions [11].

In 1970, Houk et al. introduced the concept of “*periselectivity*” as *the selective formation of one of the thermally allowed pericyclic reaction products* [56,57]. However, as the pericyclic mechanism has been recently ruled out [11], the term “periselectivity” has no sense. Therefore, in order to provide a more precise definition of the selectivity in the formation of structural isomers resulting from competitive *pseudocyclic* reactions, the “*pseudocyclic* selectivity” concept is established herein. Thus, the unexpected formation of the [3 + 2] cycloadduct **26** in Scheme 10, as well as the preferential formation of the P-AE adduct **16** *versus* the P-DA cycloadduct **15** with the increased polar character of the reaction, are examples of the proposed *pseudocyclic* selectivity. However, note that formation of the P-AE adducts **22** and **23** *versus* formation of the P-DA cycloadduct **24**, which come from the electrophilic attack of LA complex **12** to different positions of HPA **21**, is related with the chemoselectivity of this Prins reaction. 

## 4. Conclusions

The BF_3_ LA-catalyzed P-DA and P-AE reactions of 2MBD **14**, having an allylic hydrogen, with formaldehyde **20** have been studied within MEDT at the MPWB1K/6-311G(d,p) computational level. In the absence of the LA catalyst, both reactions present very high activation energies. Coordination of the BF_3_ LA to the oxygen of formaldehyde **20** drastically accelerates both reactions as a consequence of the high electrophilic character of the formed LA complex **12**, which favors these reactions to take place with very low activation enthalpy, less than 2.2 kcal·mol^−1^, and very high polar character; the P-AE reaction being slightly favored as a consequence of the larger polar character of **TS21-AE** than **TS1-DA**. The P-AE reaction between *s*-*trans* 2MBD **14** and LA complex **12** takes place through a stepwise mechanism, in which the hydrogen transfer process takes place after the formation of the first C–C single bond. However, the very similar energies found for the TSs and intermediate makes this finding experimentally irrelevant.

The Prins reaction between HPA **21** and LA complex **12** has also been studied as a computational model of the reaction experimentally reported by Künzer using steroidal olefin **17** [18]. The corresponding MEDT study, which allows explaining the experimental outcomes, supports 2MBD **14** as a computational model for this comparative study of the competitiveness between P-DA and P-AE reactions. For the reaction between HPA **21** and LA complex **12**, the corresponding P-DA reaction presents very high activation energy as the consequence of the aromatic character of the diene framework, lost along the cycloaddition reaction. For the two most favorable competitive P-AE reactions, two different mechanisms with low activation energies have been found.

Finally, an ELF topological analysis of the stationary points involved in the P-DA reaction and the P-AE reaction of *s*-*trans* 2MBD **14** with LA complex **12** allows explaining the competitive character of these polar reactions. Both **TS1-DA** and **TS21-AE**, which are associated to the nucleophilic attack of the C1 carbon of 2MBD **14** on the carbonyl C6 carbon of LA complex **12**, form a pair of stereoisomeric TSs with a very similar electronic structure [54].

The present MEDT study allows establishing the competitiveness of the P-AE reactions in P-DA reactions when the diene substrate possesses, at least, one allylic hydrogen. This competitiveness, which is a consequence of the conformational relationship between the TSs involved in the RDSs of the P-DA and P-AE reactions, is increased with the polar character of the reaction; thus, while in low polar reactions formation of the corresponding DA cycloadduct is kinetically and thermodynamically favored, in highly polar reactions, formation of the corresponding P-AE adduct may be kinetically favored. The selectivity in the formation of P-DA cycloadducts and P-AE adducts such as **15** and **16**, which constitute a pair of structural isomers coming from two stereoisomeric TSs, has been defined herein as *pseudocyclic* selectivity. 

## Figures and Tables

**Table 1:** B3LYP/6-31G(d) electronic chemical potential (μ), chemical hardness (η), global electrophilicity (ω) and global nucleophilicity (*N*), in eV, of *s*-*cis* and *s*-*trans* 2MBDs **14**, HPA **21**, formaldehyde **20** and LA complex **12**.

	μ	η	ω	*N*
**12**	−5.93	6.52	2.69	−0.07
**20**	−4.23	6.16	1.45	1.81
*s*-*trans***14**	−3.31	5.50	0.99	3.07
*s*-*cis***14**	−3.30	5.77	0.94	2.94
**21**	−2.95	6.10	0.71	3.11

## Supplementary Materials

The following are available online, Comparative study of the DA and AE reactions between 2MBD **14** and formaldehyde **20**. ELF topological analysis of the competitive P-DA and P-AE reactions between 2MBD **14** and LA complex **12**. QTAIM analysis of the electron density in the C1–C6 region at **TS1-DA** and **TS21-AE**. Tables with the MPWB1K/6-311G(d,p) total and relative energies, in gas phase and in dioxane, and with thermodynamic data of the stationary points involved in the competitive P-DA and P-AE reactions between 2-methylbutadiene **14** and LA complex **12**. Tables with the MPWB1K/6-311G(d,p) total and relative energies, in gas phase and in dioxane, as well as thermodynamic data, of the stationary points involved in the P-DA and P-AE reactions between HPA **21** and LA complex **12**.
