# Supplementary material for: A Molecular Electron Density Theory Study of the Competitiveness of Polar Diels–Alder and Polar Alder-ene Reactions
Source: Molecules. 2018 Jul 31;23(8):1913. doi: 10.3390/molecules23081913 (PMC6222353; doi:10.3390/molecules23081913)
Supplement: Supplementary file 1 [file molecules-23-01913-s001.pdf]

Supplementary Information

A Molecular Electron Density Theory Study of the Competitiveness of Polar Diels-Alder and Polar Alder Ene Reactions

Luis R. Domingo,[*a] Mar Ríos-Gutiérrez,[a] and Patricia Pérez[b]

[a] Department of Organic Chemistry, University of Valencia, Dr. Moliner 50, E-46100 Burjassot, Valencia, Spain.
[b] Universidad Andres Bello, Facultad de Ciencias Exactas, Departamento de Ciencias Químicas, Av. República 498, 8370146, Santiago, Chile.

Index

S2 1. Comparative study of the DA and AE reactions between 2MBD **14** and formaldehyde **20**.

S5 2. ELF analysis of the competitive P-DA and P-AE reactions between 2MBD **14** and LA complex **12**.

S10 3. QTAIM analysis of the electron density in the C1−C6 region at **TS1-DA** and **TS21-AE**.

S12 Table with the MPWB1K/6-311G(d,p) total and relative energies, in gas phase and in dioxane, of the stationary points involved in the more favourable C1−C6 regioisomeric pathways associated to the competitive P-DA and P-AE reactions between 2MBD **14** and LA complex **12**.

S13 Table with thermodynamic data of the stationary points involved in the competitive P-DA and P-AE reactions between 2-methylbutadiene **14** and LA complex **12**.

S14 Table with the MPWB1K/6-311G(d,p) total and relative energies, in gas phase and in dioxane, of the stationary points involved in the P-DA and P-AE reactions between HPA **21** and LA complex **12**.

S15 Table with thermodynamic data of the stationary points involved in the competitive P-DA and P-AE reactions between 2-methylbutadiene HPA **21** and LA complex **12**.

S16 MPWB1K/6-311G(d,p) gas phase total energies, the only imaginary frequency of the TSs, and Cartesian coordinates of the stationary points involved in the P-DA and P-AE reactions between 2-methylbutadiene **14** and LA complex **12**, and in the P-DA and P-AE reactions between HPA **21** and LA complex **12**.

*1. Comparative study of the DA and AE reactions between 2MBD **14** and formaldehyde **20***

Due to the non-symmetry of both reagents, these reactions can take place along two regioisomeric reaction paths related to the initial nucleophilic/electrophilic C1–C6 or C4–C6 two-center interaction. However, according to the analysis of the Parr functions (see section 3.1), only the more favourable C1–C6 regioisomeric reaction paths were studied in order to compare the two competitive DA and AE reactions. Analysis of the stationary points involved in the two DA and AE reaction paths indicates that both take place through a two-step mechanism involving the formation of a molecular complex (MC) at an early stage of the reactions (see Scheme S1). MPWB1K/6-311G(d,p) total and relative gas phase electronic energies of the stationary points involved in the two competitive reaction paths are given in Table S1.

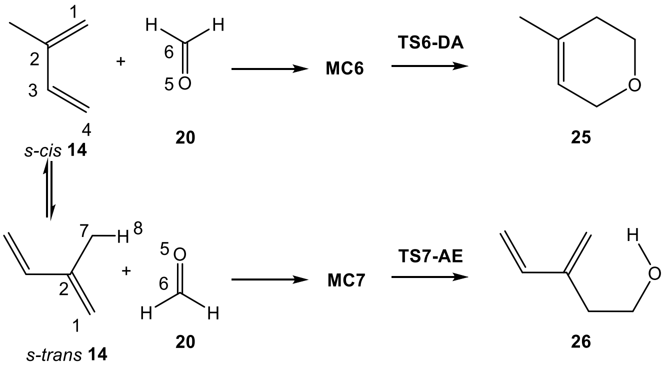

Scheme S1. Competitive DA and AE reaction paths associated to the reaction of 2MBD **14** with formaldehyde **20** along the more favourable C1–C6 regioisomeric pathways.

2MBD **14** can be found in two conformations, the *s-trans* **14** and the *s-cis* **14**. These conformations are in equilibrium due to the free bond rotation of the central C2–C3 single bond of the butadiene system. The *s-cis* conformation is 0.4 kcal·mol^{-1} higher in energy than the *s-trans* one due to the steric hindrance between the internal methylene hydrogens in the *s-cis* conformation. The gas phase activation energy associated to the interconversion of the two conformers via **TS-rot** is very low, 3.7 kcal·mol^{-1}. Interestingly, while the two conformations can be involved in the AE reaction path, only the *s-cis* one can participate in the DA reaction path. As the *s-trans* conformation is more stable than the *s-cis* one, the former was selected as the energy reference for both DA and AE reaction paths.

The first step of each one of these reaction paths is the formation of an MC in which formaldehyde **20** is located above 2MBD **14**, forming a hydrogen bond between the O5 oxygen and the H8 hydrogen at an O5–H8 distance of ca. 2.5 Å. These species, which are two minima between the reagents and the TSs of these reactions, are found 2.9 (**MC6**) and 5.5 (**MC7**) kcal·mol^{-1} below the reagents. The higher energy of **MC6** is due to the *s-cis* conformation of the 2MBD framework. From these MCs, formation of cycloadduct **25** or homoallylic alcohol **26** takes place along one elementary step *via* **TS6-DA** or **TS7-AE**. Since both MCs are in equilibrium, the activation energies associated to these reaction paths from the more stable **MC7** are 24.9 (**TS6-DA**) and 28.1 (**TS7-AE**) kcal·mol^{-1}; the overall reactions being exothermic by 38.6 (**25**) and 20.4 (**26**) kcal·mol^{-1}. The high activation energies associated to these DA and AE reactions prevent the reaction between 2MBD **14** and formaldehyde **20** to take place experimentally. These energy results suggest that, under drastic reaction conditions, the reaction of 2MBD **14** with formaldehyde **20** would predominantly give cycloadduct **25**, as **TS7-AE** is 3.1 kcal·mol^{-1} higher in energy than **TS6-DA**.

Table S1. MPWB1K/6-311G(d,p) gas phase total (E, in a.u.) and relative[a] (ΔE, in kcal·mol^{-1}) energies of the stationary points involved in the more favourable C1–C6 regioisomeric pathways associated to the competitive DA and AE reactions between 2MBD **14** and formaldehyde **20**.

	E	ΔE
s-trans **14**	-195.230549	
s-cis **14**	-195.229861	0.4
**TS-rot**	-195.224585	3.7
**20**	-114.472956	
**MC6**	-309.708169	-2.9
**TS6-DA**	-309.672509	19.4
**25**	-309.764994	-38.6
**MC7**	-309.712348	-5.5
**TS7-AE**	-309.667498	22.6
**26**	-309.736053	-20.4

[a] Relative to the more favourable *s-trans* conformation of 2MBD **14**.

The geometries of the TSs involved in the more favourable C1–C6 regioisomeric pathways associated to the competitive DA and AE reactions between 2MBD **14** and formaldehyde **20**, including the distances between the nuclei involved in the formation of the new C1–C6 and O5–C4(H8) bonds, are displayed in Figure S1. Considering that formation of C–C single bonds [1] takes place at the short distance range of 2.0 – 1.9 Å,

prior to the O–C(H) ones [2], these geometrical parameters indicate that at **TS6-DA** and **TS7-AE** formation of the C–C single bond is more advanced than formation of the O–C single bond (DA) and the hydrogen transfer process (AE). Note that the short C1–C6 distance of ca. 1.86 Å at **TS7-AE** suggests that the C1–C6 may be already formed.

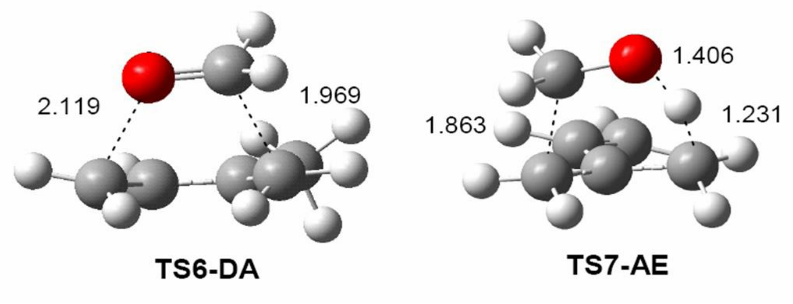

Figure S1. MPWB1K/6-311G(d,p) gas phase optimised geometries of the TSs involved in the more favourable C1–C6 regioisomeric pathways associated to the competitive DA and AE reactions between 2MBD **14** and formaldehyde **20**. Distances are given in angstroms, Å.

Thorough studies have made it possible to establish a good correlation between the polar character of a reaction and its feasibility; the more polar the reaction, i.e. the higher the GEDT [1] at the most favourable TS, the faster the reaction. Cycloadditions with GEDT values near 0.0 e correspond to non-polar processes, whereas values higher than 0.2e correspond to polar processes. The GEDT computed at more favourable C1–C6 regioisomeric TSs involved in the competitive DA and AE reactions between 2MBD **14** and formaldehyde **20** is 0.15 e at **TS6-DA** and 0.29 e at **TS7-AE**. These values indicate that while the P-DA reaction has only some polar character, which accounts for the high computed activation energy, the AE reaction has a strong polar character. Note, however, that the stronger polar character of **TS7-AE** is just a consequence of the fact that the C1–C6 bond formation is more advanced.

*2. ELF analysis of the competitive P-DA and P-AE reactions between 2MBD **14** and LA complex **12**.*

In order to compare the C–C bond-formation process along the competitive P-DA and P-AE reactions between 2MBD **14** and LA complex **12**, a topological analysis of the electron localisation function (ELF) [3] of the stationary points involved in the two reaction paths was carried out. The population of the most relevant ELF valence basins of the corresponding stationary points are given in Table S2 while ELF valence basin attractor positions are shown in Figure S2.

ELF topological analysis at the *s-trans* and *s-cis* conformations of 2MBD **14** provides a similar bonding pattern for both conformers. For *s-trans* 2MBD **14**, the topological analysis of the ELF shows the presence of two pairs of disynaptic basins, V(C1,C2) and V'(C1,C2), and V(C3,C4) and V'(C3,C4), integrating total populations of 3.41 e and 3.37 e, respectively, and one V(C2,C3) disynaptic basin, integrating 2.22 e, related to the C1–C2 and C3–C4 double bonds, and the C2–C3 single bond of 2MBD **14**. On the other hand, ELF topological analysis of LA complex **12** shows the presence of two disynaptic basins, V(O5,C6) and V'(O5,C6), integrating a total population of 2.51 e, which are associated with a carbonyl O5–C6 single bond, and two monosynaptic basins, V(O5) and V'(O5), integrating a total electron density of 5.05 e, which are associated with the non-bonding electron density of the O5 oxygen, one of them facing the boron center of BF_3. This analysis clearly shows a polarisation of the O5−C6 bonding region by the presence of the BF_3 LA.

The reaction path associated with the P-DA reaction begins with the formation of **MC1**, d(C1–C6) = 2.646 Å and d(C4–O5) = 3.160 Å. At **MC1**, while the population of the two pairs of disynaptic basins, V(C1,C2) and V'(C1,C2), and V(C3,C4) and V'(C3,C4), present in *s-cis* 2MBD **14**, decrease by 0.14 e and 0.08 e, respectively, the population of the V(C2,C3) disynaptic basin increases by 0.08 e. On the other hand, at the LA complex **12** framework, while the total population of the V(O5,C6) disynaptic basins decreases by only 0.09 e, that of the two V(O5) and V'(O5) monosynaptic basins increases by 0.13 e. This analysis shows no relevant changes in the electron populations with respect to the separated reagents. At **MC1**, the GEDT is practically null.

At **TS1-DA**, d(C1–C6) = 1.942 Å and d(C4–O5) = 2.890 Å, it is worth mentioning that a new V(C1) monosynaptic basin with a population of 0.63 e is observed. This significant topological change indicates that, at the C–C distance of

1.942 Å, the first C1–C6 single bond has not been yet formed [1]. Moreover, the two V(C1,C2) and V'(C1,C2) disynaptic basins present at **MC1** have merged into one V(C1,C2) disynaptic basin, showing a decrease of the population to 2.73 e. The V(C2,C3) disynaptic basin continues increasing its population to 2.36 e, whereas the two V(C3,C4) and V'(C3,C4) disynaptic basins continue decreasing their population to 3.19 e. The population of the V(O5,C6) disynaptic basin strongly decreases to 1.86 e, while the population of the two V(O5) and V'(O5) monosynaptic basins integrate 5.80 e, showing the strong polarisation of the electron density in the activated formaldehyde framework. At **TS1-DA**, the GEDT reaches a value of 0.40 e, emphasising the high polar character of this P-DA reaction.

At cycloadduct **25**, d(C1–C6) = 1.484 Å and d(C4–O5) = 1.432 Å, while the population of the V(C1,C6) disynaptic basin is 1.95 e, the population of the V(C4,O5) disynaptic basin, associated to the second C4–O5 single bond formed at the end of the reaction, is 1.34 e. Also note that the population of the V(C1,C2) disynaptic basin reaches 2.01 e, that of the V(C2,C3) and V'(C2,C3) disynaptic basins integrate 3.60 e, and that of the V(C3,C4) disynaptic basin reaches 2.06 e. On the other hand, the population of V(O5,C6) disynaptic basin has decreased to 1.34 e, while the total population of the V(O5) and V'(O5) monosynaptic basins is 4.82 e.

The reaction path associated with the polar P-AE reaction begins with the formation of **MC2**, d(C1–C6) = 2.716 Å, d(C7–H8) = 1.089 Å and d(O5–H8) = 2.855 Å. At **MC2**, while the population of the two pairs of disynaptic basins, V(C1,C2) and V'(C1,C2), and V(C3,C4) and V'(C3,C4), present in *s-trans* 2MBD **14**, decreases by 0.13 e and 0.01 e, respectively, the population of the V(C2,C3) disynaptic basin increases only by 0.02 e. The population of the V(C7,H8) protonated basin integrates 1.97 e. On the other hand, the ELF picture for the LA complex **12** framework also shows one V(O5,C6) disynaptic basin integrating 2.44 e, and two V(O5) and V'(O5) monosynaptic basins integrating a total electron density of 5.13 e. Again, this analysis shows a polarisation along the O5–C6 bonding region by the presence of BF_3 LA. At **MC2**, the GEDT is null.

At **TS21-AE**, d(C1–C6) = 1.806 Å d(C7–H8) = 1.099 Å and d(O5–H8) = 2.115 Å, a new V(C1,C6) disynaptic basin with an initial population of 0.94 e is observed. This significant topological change indicates that the new C1–C6 single bond has been

already formed at a C–C distance of 1.806 Å [1]. Note that the only topological difference between **TS1-DA** and **TS21-AE** is the presence of the V(C1) monosynaptic basin at **TS1-DA** and the presence of the V(C1,C6) disynaptic basin at **TS21-AE**, because of the more advanced character of the later. Besides, the two V(C1,C2) and V'(C1,C2) disynaptic basins present at **MC2** have merged into one V(C1,C2) disynaptic basin, which suffers a strong decreasing of the population to 2.53 e, the population of the V(C2,C3) disynaptic basin slightly increases to 2.31 e, while the total population of the two V(C3,C4) and V'(C3,C4) disynaptic basins decrease to 3.26 e. The electron population of the V(C2,C7) slightly increases to 2.10 e. On the other hand, the population of V(O5,C6) disynaptic basin strongly decreases to 1.68 e, while that of the two monosynaptic basins, V(O5) and V'(O5), notably increase to 6.0 e. These behaviours point out a similar C1−C6 single bond formation pattern along the two P-DA and P-AE competitive reaction paths. Finally, the population of the V(C7,H8) protonated basin decreases to 1.89 e. At **TS21-AE**, the GEDT is 0.49 e.

At **IN2**, d(C1–C6) = 1.709 Å, d(C7–H8) = 1.107 Å and d(O5–H8) = 2.021 Å, while the population of the V(C1,C6) disynaptic basin has increased by 0.28 e, and that of the V(C1,C2) disynaptic basin has decreased by 0.15 e, the population corresponding to the V(C2,C3) and V(C3,C4) disynaptic basins practically remain unchanged, and that of the V(O5,C6) disynaptic basin has decreased by 0.08 e. The population of the V(C7,H8) protonated basin is 1.86 e. The total population of the V(O5) and V'(O5) monosynaptic basins remains unchanged. At **IN2**, the GEDT is 0.60 e.

At **TS22-AE**, d(C1–C6) = 1.608 Å d(C7–H8) = 1.191 Å and d(O5–H8) = 1.565 Å, the population of the V(C1,C2) disynaptic basin decreases by 0.18 e, while that of the V(C2,C7) disynaptic basin increases by 0.18 e. On the other hand, while the population of the V(C1,C6) disynaptic basin increases to 1.58 e, the population of the V(C7,H8) protonated basin decreases by 0.23 e, which is related with the next transfer of the H8 hydrogen from the 2MBD C7 carbon towards the formaldehyde O5 oxygen. As a consequence, the population of the V(O5,C6) disynaptic basin also slightly decreases to 1.52 e. The GEDT at **TS22-AE** is the highest found, 0.64 e.

Finally, at the AE adduct **16**, d(C1–C6) = 1.515 Å d(C7–H8) = 2.347 Å and d(O5–H8) = 0.966 Å, the most relevant topological change is the formation of one V(O5,H8) protonated basin, which integrates 1.83e, being associated with the formation of the new O5–H8 bond. Moreover, two V(C2,C7) and V'(C2,C7) disynaptic basins

integrating a total population of 3.42e, associated with the new C2−C7 double bond, appear at the butadiene framework.

Table S2. ELF valence basin populations of the stationary points involved in the competitive P-DA and P-AE reactions between 2MBD **14** and LA complex **12**. Distances are given in angstroms, Å, while GEDT values and electron populations are displayed in average number of electron, e.

Points	*s-trans* **14**	*s-cis* **14**	**12**	**MC1**	**TS1-DA**	**15**	**MC2**	**TS21-AE**	**IN2**	**TS22-AE**	**16**
d(C1-C6)				2.646	1.942	1.484	2.716	1.806	1.709	1.608	1.515
d(C7-H8)				3.160	2.890	1.432	1.089	1.099	1.107	1.191	2.347
d(O5-H8)							2.855	2.115	2.021	1.565	0.966
GEDT				0.07	0.40	0.39	0.06	0.49	0.60	0.64	0.53
V(C1,C2)	1.63	1.67		1.78	2.73	2.01	1.79	2.53	2.38	2.20	2.01
V'(C1,C2)	1.78	1.77		1.52			1.49				
V(C2,C3)	2.22	2.16		2.24	2.36	1.82	2.24	2.31	2.33	2.29	2.21
V’(C2,C3)						1.78					
V(C3,C4)	1.61	1.64		1.63	1.63	2.06	1.69	1.66	1.69	1.75	1.77
V’(C3,C4)	1.76	1.79		1.72	1.56		1.67	1.60	1.53	1.50	1.62
V(C2,C7)	2.00	1.98		2.02	2.07	2.02	2.00	2.10	2.13	2.31	1.85
V’(C2,C7)											1.57
V(O5,C6)			1.19	2.42	1.86	1.34	2.44	1.68	1.60	1.52	1.38
V’(O5,C6)			1.32								
V(O5)			2.82	2.99	3.70	2.38	2.94	3.95	4.05	2.00	2.13
V’(O5)			2.23	2.19	2.12	2.44	2.19	2.05	1.95	4.06	2.39
V(C1)					0.63						
V(C1,C6)						1.95		0.94	1.22	1.58	1.91
V(C4,O5)						1.34					
V(C7,H8)	2.12	1.99		1.98	1.97	1.99	1.97	1.89	1.86	1.63	
V(O5,H8)											1.83

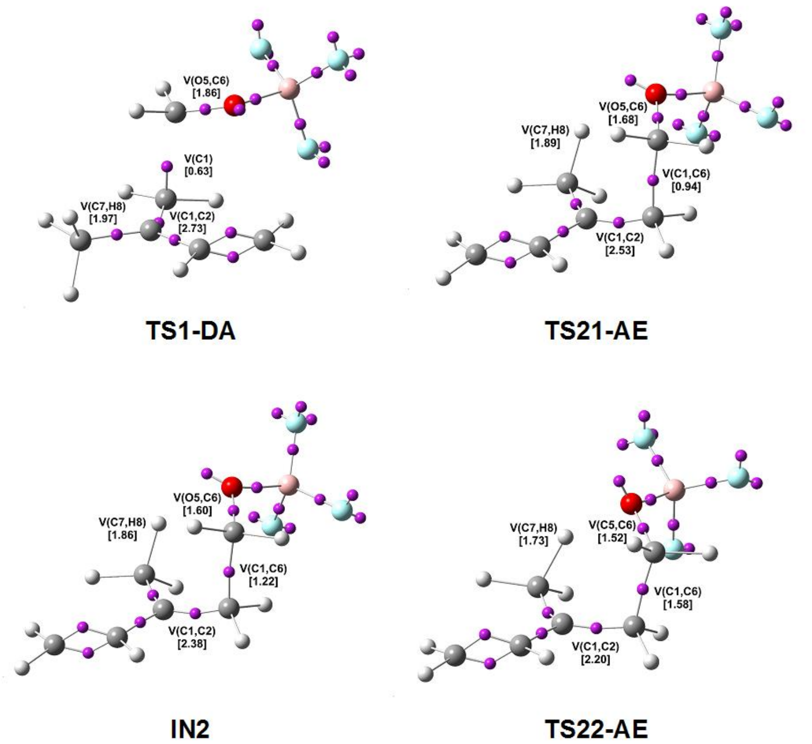

Figure S2. Attractor positions of the ELF valence basins for the TS involved in the P-DA reaction, **TS1-DA**, and TSs and intermediate involved in the P-AE reaction, **TS21-AE**, **IN2** and **TS22-AE**, between 2MBD **14** and LA complex **12**. The electron populations, in average number of electrons, e, are given in brackets.

*3. QTAIM analysis of the electron density in the C1−C6 region at **TS1-DA** and **TS21-AE**.*

In order to confirm the bonding pattern arising from the topological analysis of the ELF between the C1 and C6 carbons at **TS1-DA** and **TS21-AE**, a QTAIM topological analysis of the electron density distribution at both TSs was performed. The calculated Quantum Theory of Atoms in Molecules (QTAIM) [4] parameters of the critical points (cps) found in the C1–C6 region are gathered in Table S3, while the contour line maps of the Laplacian of the electron density in the molecular plane defined by the C1, C6 and O5 nuclei are represented in Figure S3.

Table S3. QTAIM parameters (in a.u.), namely, the electron density ρ_{cp} and its Laplacian $\nabla^2\rho_{cp}$, of the cp associated with the C1–C6 region at **TS1-DA** and **TS21-AE**.

	cp	ρ_{cp}	$\nabla^2\rho_{cp}$
**TS1-DA**	1	$9.08 \cdot 10^{-2}$	$1.13 \cdot 10^{-3}$
**TS21-AE**	2	$1.22 \cdot 10^{-1}$	$-7.26 \cdot 10^{-2}$

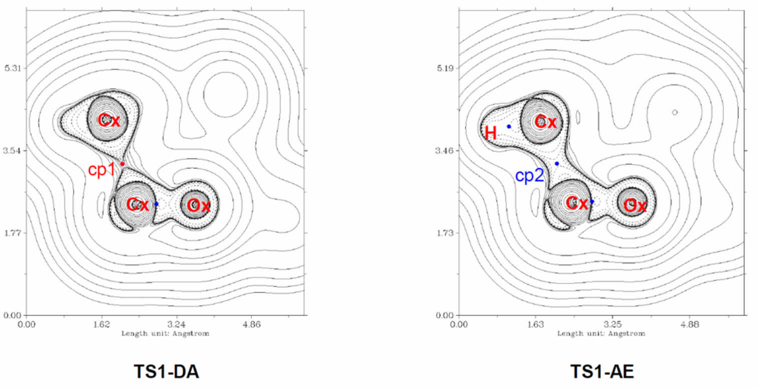

Figure S3. Representations of the contour line maps of the Laplacian of the electron density of **TS1-DA** and **TS21-AE** on the C1–C6–O5 molecular plane. Cps with $\nabla^2\rho_{cp} < 0$ are coloured in blue and cps with $\nabla^2\rho_{cp} > 0$ are coloured in red.

In the QTAIM framework, values of electron density less than 0.1 a.u. and positive values of the Laplacian of the electron density indicates that the trajectories of the gradient paths involving the considered cp are not associated to covalent bonds. As shown in Table S3, the electron density in the C1–C6 region at **TS21-AE**, $9.08 \cdot 10^{-2}$

a.u., is closer to that at **TS1-DA**, 1.22·10^{-1} a.u., while the Laplacian of the electron density at cp1 (**TS1-DA**) is positive, 1.13·10^{-3}, but negative at cp2 (**TS21-AE**), −7.26·10^{-2}, though very close to zero in any case (see Table S3). Interestingly, while the contour lines of the Laplacian of the electron density in the C1−C6 region are separated in **TS1-DA**, they are linked at **TS21-AE** (see Figure S3).

These results indicate that while cp2 can be associated to a formed, or proximate to be formed, C1−C6 single bond at **TS21-AE**, cp1 indicates that the formation of the C1−C6 single bond does not begun at that **TS1-DA**, in agreement with the topological analysis of the ELF at both TSs.

References

1. Domingo, L.R. A new C-C bond formation model based on the quantum chemical topology of electron density. *RSC Adv.* **2014**, *4*, 32415-32428
2. Ríos-Gutiérrez, M. ; Pérez, P.; Domingo, L. R. A bonding evolution theory study of the mechanism of [3+2] cycloaddition reactions of nitrones with electron-deficient ethylenes. *RSC Adv.* 2015, 58464–58477.
3. Becke, A.D.; Edgecombe, K.E. A simple measure of electron localization in atomic and molecular-systems. *J. Chem. Phys.* **1990**, *92*, 5397-5403.
4. Bader, R. F. W. *Atoms in Molecules. A Quantum Theory*; Claredon Press: Oxford, U.K., 1990

Table S4. MPWB1K/6-311G(d,p) total (E, in a.u.) and relative[a] (ΔE, in kcal·mol^{-1}) energies, in gas phase and in dioxane, of the stationary points involved in the more favourable C1–C6 regioisomeric pathways associated to the competitive P-DA and P-AE reactions between 2MBD **14** and LA complex **12**.

	E	ΔE	E	ΔE
	Gas phase		*Dioxane*	
s-trans **14**	-195.230549		-195.231527	
s-cis **14**	-195.229861	0.4	-195.230781	0.5
**TS-rot**	-195.224585	3.7	-195.225441	3.8
**12**	-439.042570		-439.049312	
**MC1**	-634.284438	-7.1	-634.290736	-6.2
**TS1-DA**	-634.280126	-4.4	-634.289377	-5.4
**15**	-634.347268	-46.5	-634.352532	-45.0
**MC2**	-634.289211	-10.1	-634.295246	-9.0
**TS21-AE**	-634.279619	-4.1	-634.290196	-5.9
**IN2**	-634.279717	-4.1	-634.293154	-7.7
**TS22-AE**	-634.277732	-2.9	-634.289624	-5.5
**16**	-634.318603	-28.5	-634.324196	-27.2

[a] Relative to the more favourable *s-trans* conformation of 2MBD **14**.

Table S5. Enthalpies (H, in a.u.), entropies (S, in cal·mol^{-1}·K^{-1}) and Gibbs free energies (G, in a.u.), and relative[a] enthalpies (ΔH, in kcal·mol^{-1}), entropies (ΔS, in cal·mol^{-1}·K^{-1}), and Gibbs free energies (ΔG, in kcal·mol^{-1}), computed at -5 ºC in dioxane, for the stationary points involved in the competitive P-DA and P-AE reactions between 2-methylbutadiene **14** and LA complex **12**.

	H	ΔH	S	ΔS	G	ΔG
s-trans **14**	-195.110884		70.1		-195.140826	
s-cis **14**	-195.109512	0.9	72.7	2.6	-195.140569	0.2
**TS-rot**	-195.105246	3.5	70.0	-0.1	-195.135152	3.6
**12**	-438.999226		76.1		-439.031725	
**MC1**	-634.116988	-4.3	111.4	-34.7	-634.164608	5.0
**TS1-DA**	-634.115687	-3.5	104.1	-42.0	-634.160173	7.8
**15**	-634.173539	-39.8	96.7	-49.5	-634.214842	-26.5
**MC2**	-634.121190	-7.0	108.4	-37.8	-634.167493	3.2
**TS21-AE**	-634.116320	-3.9	102.9	-43.3	-634.160274	7.7
**IN2**	-634.118350	-5.2	102.1	-44.0	-634.161996	6.6
**TS22-AE**	-634.117997	-4.9	98.4	-47.7	-634.160039	7.9
**16**	-634.146916	-23.1	103.1	-43.0	-634.190981	-11.6

[a] Relative to the more favourable *s-trans* conformation of 2-methylbutadiene *s-trans* **12**.

Table S6. MPWB1K/6-311G(d,p) total (E, in a.u.) and relative[a] (ΔE, in kcal·mol^{-1}) energies, in gas phase and in dioxane, of the stationary points involved in the polar P-DA and P-AE reactions between HPA **21** and LA complex **12**.

	E	ΔE	E	ΔE
	Gas phase		*Dioxane*	
**21**	-542.966919		-542.968618	
**12**	-439.042584		-439.048743	
**MC3**	-982.024419	-9.4	-982.030402	-8.2
**TS3-AE**	-982.006664	1.8	-982.018790	-0.9
**22**	-982.055752	-29.0	-982.061809	-27.9
**MC4**	-982.023346	-8.7	-982.029072	-7.3
**TS41-AE**	-982.008797	0.4	-982.017291	0.0
**IN4**	-982.010363	-0.5	-982.020614	-2.0
**TS42-AE**	-982.008916	0.4	-982.019112	-1.1
**23**	-982.064134	-34.3	-982.069490	-32.7
**MC5**	-982.021859	-7.8	-982.027473	-6.3
**TS5-DA**	-981.985646	15.0	-981.994161	14.6
**25**	-982.005171	2.7	-982.011112	3.9

[a] Relative to **21** + **12**.

Table S7. Enthalpies (H, in a.u.), entropies (S, in cal·mol^{-1}·K^{-1}) and Gibbs free energies (G, in a.u.), and relative[a] enthalpies (ΔH, in kcal·mol^{-1}), entropies (ΔS, in cal·mol^{-1}·K^{-1}), and Gibbs free energies (ΔG, in kcal·mol^{-1}), computed at -5 ºC in dioxane, for the stationary points involved in the competitive P-DA and P-AE reactions between HPA **21** and LA complex **12**.

	H	ΔH	S	ΔS	G	ΔG
**21**	-542.689516		96.5		-542.730739	
**12**	-438.999695		72.7		-439.030775	
**MC3**	-981.699608	-6.5	126.9	-42.3	-981.753855	4.8
**TS3-AE**	-981.689026	0.1	124.3	-44.9	-981.742145	12.2
**22**	-981.728024	-24.4	122.5	-46.7	-981.780385	-11.8
**MC4**	-981.698701	-6.0	129.2	-40.0	-981.753917	4.8
**TS41-AE**	-981.686094	2.0	123.7	-45.5	-981.738946	14.2
**IN4**	-981.688643	0.4	124.9	-44.3	-981.742009	12.2
**TS42-AE**	-981.688620	0.4	119.1	-50.1	-981.739516	13.8
**23**	-981.735702	-29.2	120.4	-48.8	-981.787135	-16.1
**MC5**	-981.697291	-5.1	131.4	-37.8	-981.753454	5.1
**TS5-DA**	-981.663047	16.4	122.3	-46.9	-981.715289	29.0
**25**	-981.677182	7.5	124.1	-45.1	-981.730202	19.6

[a] Relative HPA **21** and LA complex **12**.

MPWB1K/6-311G(d,p) gas phase total energies, the only imaginary frequency of the TSs, and Cartesian coordinates of the stationary points involved in the P-DA and P-AE reactions between 2-methylbutadiene **14** and LA complex **12**, and in the P-DA and P-AE reactions between HPA **21** and LA complex **12**.

s-trans **14**

E(RmPWB95) = -195.230548603 au

C	0.63073800	1.68364900	0.00000000
H	0.09478000	2.61944500	0.00000000
H	1.70786200	1.73545300	0.00000000
C	0.00000000	0.51386800	0.00000000
C	-1.49829000	0.44598700	0.00000000
H	-1.90352200	0.94212900	0.87670600
H	-1.90352200	0.94212900	-0.87670600
H	-1.86853800	-0.57099100	0.00000000
C	0.79493400	-0.70883700	0.00000000
C	0.35042300	-1.95674100	0.00000000
H	1.86423900	-0.54596900	0.00000000
H	1.03974100	-2.78450700	0.00000000
H	-0.69787200	-2.20523700	0.00000000

s-cis **14**

E(RmPWB95) = -195.229860681 au

C	-0.58335000	1.42719000	-0.09372500
H	-1.53940000	1.89749200	0.07242300
H	0.25726700	2.06948100	-0.30029400
C	-0.45160400	0.10843900	-0.06425800
C	-1.61244500	-0.80622800	0.15127400
H	-1.46069100	-1.42311900	1.03441100
H	-2.53734800	-0.25320500	0.27123100
H	-1.72714200	-1.48479500	-0.69236500
C	0.84671600	-0.54386700	-0.26406200
C	2.00088700	-0.10199800	0.20612800
H	0.82813300	-1.47386200	-0.81819600
H	2.92282800	-0.62534900	0.01310900
H	2.05513300	0.79214000	0.80753900

TS-rot

E(RmPWB95) = -195.224576525 au

1 imaginary frequencies -137.3678 cm-1

C	-1.17660300	-1.22981400	-0.15718600
C	-0.48752700	-0.12772800	0.08472200
C	0.93817300	-0.18308800	0.47808200
C	1.94245300	0.08236700	-0.33516800
H	-2.21885700	-1.18741600	-0.43255500
H	-0.72038900	-2.20428400	-0.09169100
C	-1.09050100	1.23592400	0.00183900
H	-0.56243900	1.84036700	-0.73265000
H	-2.13946000	1.18981900	-0.27162300
H	-1.00127700	1.75537900	0.95399500
H	1.14564300	-0.44160000	1.50866900
H	2.96551400	0.03820100	0.00126300
H	1.77529400	0.34356500	-1.36913800

12
E(RmPWB95) = -439.042583590 au

C	-0.00408900	0.02314400	-0.00270300
O	0.00019500	-0.09448400	1.19438900
H	0.92668200	0.16299700	-0.55063600
H	-0.95301500	-0.01205700	-0.53572600
F	1.50845500	-1.25417500	2.64715800
F	2.40115000	0.14772600	1.09343800
F	1.29156600	1.00580400	2.88434800
B	1.51741000	-0.04267800	2.09488700

MC1
E(RmPWB95) = -634.284437583 au

C	0.13694300	-0.91773500	-1.11250400
O	0.89673500	0.02501500	-0.99665900
C	-1.31488700	-0.71051900	1.08101900
H	-1.37863900	-1.73330800	1.41708300
H	-0.50038400	-0.11956100	1.46859700
C	-2.25858100	-0.19397800	0.28861500
C	-3.41419300	-1.01732800	-0.17688100
H	-3.47581200	-1.02908200	-1.26405800
H	-3.35271400	-2.03898300	0.18000400
H	-4.34698800	-0.58598100	0.18068000
H	-0.68229200	-0.83845500	-1.81929600
H	0.32562000	-1.85042500	-0.59253700
F	1.69605000	0.89242100	1.02351500
F	2.08538900	-1.30112500	0.57729700
F	3.19947400	0.30727900	-0.59657900
B	2.11419100	-0.02275400	0.11405600
C	-2.25300800	1.19580000	-0.17562200
C	-1.27792100	2.07689500	-0.00524700
H	-3.13673600	1.50074500	-0.71921200
H	-1.37293700	3.07555100	-0.39827100
H	-0.36229400	1.84716900	0.51561900

TS1-DA
E(RmPWB95) = -634.280126137 au
1 imaginary frequencies -235.0391 cm-1

C	-0.09834200	-1.12652300	-0.65756900
O	0.67627000	-0.13880500	-0.86894500
C	-1.17594700	-0.77461800	0.91930900
H	-1.41354300	-1.76131700	1.28427400
H	-0.39597100	-0.25258800	1.44876200
C	-2.15464000	-0.09747200	0.23675300
C	-3.36987700	-0.81493800	-0.22749800
H	-3.48937800	-0.71447800	-1.30434700
H	-3.35564700	-1.86500100	0.03727700
H	-4.24743300	-0.35626500	0.22483800
H	-0.83400800	-1.32127500	-1.42823400
H	0.32501700	-2.00279000	-0.17698600
F	1.56154700	0.58609300	1.17834300
F	2.38562500	-1.30471800	0.23058400
F	2.81481800	0.73348500	-0.72352500

B	1.95482200	-0.02452000	-0.01046300
C	-2.02722800	1.27281000	-0.17333800
C	-0.96701100	2.03587300	0.09291900
H	-2.82713000	1.66503300	-0.78344700
H	-0.90546600	3.03455000	-0.30774900
H	-0.14035400	1.70262700	0.69742100

15

E(RmPWB95) = -634.347267901 au

C	-0.15814700	-0.94322600	-1.26680000
O	0.64236400	0.17114400	-0.85464400
C	-1.22344000	-1.21411900	-0.24203000
H	-1.91390400	-1.95531600	-0.64147200
H	-0.78086800	-1.63875800	0.65709400
C	-1.95932700	0.04256600	0.09746600
C	-3.27150100	-0.10689600	0.78067200
H	-3.71771200	0.85551400	1.00549100
H	-3.96756600	-0.67722100	0.16831500
H	-3.14965600	-0.65192500	1.71487900
H	-0.58856800	-0.67041500	-2.22619800
H	0.53220400	-1.76388300	-1.39380800
F	0.89384400	0.03006200	1.50317700
F	2.15594100	-1.30529600	0.15665700
F	2.55022100	0.95132200	0.25283600
B	1.67750400	-0.06586700	0.40030700
C	-1.43093700	1.21975300	-0.19133600
C	-0.08612100	1.39970100	-0.79343900
H	-1.96297100	2.13111800	0.03646000
H	-0.13243100	1.79566900	-1.80657900
H	0.53182100	2.06393700	-0.19979000

MC2

E(RmPWB95) = -634.289211256 au

C	-0.24265600	-0.99798600	-1.01079600
O	-0.96405100	-0.02147700	-1.04733400
C	1.08602800	-0.72117000	1.35321400
H	0.16574800	-0.34489000	1.77179600
H	1.37586000	-1.72901100	1.61052800
C	1.86364100	0.04174000	0.57853200
C	1.49010000	1.44681600	0.25113200
H	2.26033100	2.13243500	0.59616100
H	1.39429400	1.58624900	-0.82460900
H	0.54660100	1.71421300	0.70891300
H	-0.50473000	-1.85651000	-0.40166800
H	0.63384200	-1.02650600	-1.65155900
F	-2.31326600	-1.13233800	0.56565100
F	-1.77997400	1.05422600	0.86355400
F	-3.25946600	0.45418600	-0.77438400
B	-2.23433500	0.10713700	0.01138100
C	3.10150400	-0.50447300	0.03419700
C	3.91855200	0.14359600	-0.78343600
H	3.34475000	-1.51394400	0.33602100
H	4.82017800	-0.31593200	-1.15206700
H	3.71856100	1.15422300	-1.10218400

TS21-AE

E(RmPWB95) =   -634.279619105 au

1 imaginary frequencies -144.8645 cm-1

C	0.10732000	-1.16522200	0.65256300
O	0.85799200	-0.16245900	0.97632900
C	-0.76314500	-0.86517400	-0.90433900
H	0.07155200	-0.48949100	-1.47695300
H	-1.12580100	-1.84288500	-1.18641800
C	-1.69871500	0.03997000	-0.42423900
C	-1.28865700	1.42751100	-0.21226900
H	-2.08143000	2.08659500	0.11107000
H	-0.50029000	1.38802500	0.55295000
H	-0.77927200	1.82088000	-1.08420900
H	0.61902500	-2.03505000	0.24947100
H	-0.65769300	-1.40205900	1.38428200
F	2.37924700	-1.03869800	-0.57442900
F	1.57794100	1.05910700	-0.87148400
F	3.03225600	0.64352000	0.84239800
B	2.04826100	0.14287500	0.06274600
C	-2.98167800	-0.46866600	0.00232200
C	-3.97751400	0.27377500	0.47366300
H	-3.12485200	-1.53377700	-0.10612300
H	-4.91473200	-0.17776000	0.75325800
H	-3.90240600	1.34231000	0.58373700

IN2

E(RmPWB95) = -634.279717098 au

C	0.11198700	-1.19006200	0.63911700
O	0.86956800	-0.17177300	0.97796600
C	-0.73462700	-0.90175500	-0.82077900
H	0.06634600	-0.52625000	-1.44212500
H	-1.12816500	-1.86227600	-1.12399400
C	-1.68816800	0.03064300	-0.38050600
C	-1.25852800	1.40054300	-0.15890100
H	-0.46286900	1.30969800	0.60438600
H	-0.71328000	1.78679800	-1.01281300
H	-2.02920500	2.07785000	0.17914100
H	0.65671600	-2.06390900	0.28784400
H	-0.61739400	-1.44291500	1.40341200
F	2.38911200	-1.02470000	-0.58349700
F	1.53608100	1.05009900	-0.89531500
F	3.01587400	0.69064000	0.80835800
B	2.03005900	0.15073700	0.05400600
C	-2.98536100	-0.45942000	0.01026500
C	-4.00173900	0.31285300	0.38303300
H	-3.12805100	-1.52781300	-0.05402200
H	-4.95754300	-0.11760800	0.63154900
H	-3.92438400	1.38575300	0.43358100

TS22-AE

E(RmPWB95) = -634.277731788 au

1 imaginary frequencies -454.0232 cm-1

C	0.14004800	1.30454300	-0.48829700
O	0.75279700	0.15641100	-0.86095100
C	-0.82844800	1.10428900	0.77983800
H	-0.16936400	0.82957500	1.59192900

H	-1.33520700	2.04531000	0.95639700
C	-1.71220800	0.03269200	0.39105100
C	-1.14653200	-1.25382700	0.33427800
H	-0.30462600	-0.91679900	-0.43931200
H	-0.47419900	-1.50538500	1.14549900
H	-1.74377300	-2.06541800	-0.05140200
H	0.85929400	2.04403600	-0.14537800
H	-0.44613700	1.70671400	-1.31082400
F	2.85321100	0.86958800	-0.12621100
F	1.59565500	-0.43930400	1.24237300
F	2.49087000	-1.33756200	-0.65351300
B	2.00762000	-0.20656300	-0.07909000
C	-2.99080800	0.36610700	-0.18790300
C	-3.95925600	-0.52262300	-0.38220700
H	-3.16768200	1.40926200	-0.40302100
H	-4.91671900	-0.22414200	-0.77540400
H	-3.83646800	-1.56321000	-0.12983200

16

E(RmPWB95) = -634.318603152 au

C	-0.08882100	-1.40093000	-0.16408000
O	-0.80218700	-0.33035100	-0.79747700
C	0.83849800	-0.86855700	0.90856600
H	0.24272300	-0.41955600	1.69438500
H	1.38779900	-1.70741700	1.32846400
C	1.77665400	0.14612000	0.32387800
C	1.46875700	1.44115100	0.35158200
H	-0.19574300	0.40725000	-0.94134600
H	0.60103300	1.79162200	0.88907300
H	2.08794000	2.17456600	-0.13974900
H	-0.85380900	-2.05068500	0.23856400
H	0.45013000	-1.92394900	-0.94767800
F	-2.89191500	-0.91768600	0.08283900
F	-1.72985000	0.75092300	1.10436400
F	-2.62745000	1.11351600	-0.95364000
B	-2.17168000	0.21162100	-0.06766700
C	2.97301100	-0.37447500	-0.34201400
C	4.13918000	0.24650600	-0.39526400
H	2.88394200	-1.36009700	-0.78118600
H	4.98466900	-0.19128900	-0.89882700
H	4.28648100	1.20458500	0.07837600

21

E(RmPWB95) = -542.966919057 au

C	3.43939400	-0.17013000	-0.34042200
C	2.77358200	0.77148400	0.65314500
C	1.30911900	0.85664200	0.37868900
C	0.60410200	-0.46040800	0.41471100
C	1.26563300	-1.38909700	-0.60897000
C	2.75257800	-1.52163700	-0.34395900
C	0.70608700	1.98407700	0.04516400
C	-0.88109000	-0.36266100	0.22116400
C	-1.50380800	0.82630800	-0.12339500
C	-0.73323100	2.09736600	-0.29860400
C	-2.88113500	0.84404300	-0.29672200
H	-3.36001600	1.77405200	-0.56464300
C	-3.64029000	-0.29053100	-0.12798100

C	-3.02221600	-1.47486500	0.23013900
C	-1.65752700	-1.50062800	0.40139800
H	4.49610200	-0.27969100	-0.10702600
H	3.22391600	1.75972200	0.61352500
H	1.09830100	-0.97664000	-1.60385700
H	2.89866300	-1.99937500	0.62655400
H	-0.83395300	2.44263200	-1.32986100
H	-4.70853800	-0.25327000	-0.26637400
H	-3.60342000	-2.37040300	0.37809100
H	-1.17405800	-2.42230700	0.68913200
H	3.20793100	-2.17529900	-1.08435400
H	3.37547200	0.26895900	-1.33582700
H	2.92957100	0.38429900	1.66256700
H	0.78603300	-0.90411400	1.39912900
H	-1.19428600	2.87874500	0.30731900
H	0.78752000	-2.36563800	-0.58622900
H	1.28779800	2.89498300	0.00494600

MC3
E(RmPWB95) = -982.024419389 au

C	0.04018200	-0.08340600	0.19466900
C	-0.08703700	-0.52156700	-1.26044400
C	1.20072800	-0.24487500	-1.95601500
C	2.37800100	-0.94544800	-1.36475100
C	2.52798800	-0.45788000	0.07890800
C	1.25045700	-0.71747000	0.85700400
C	1.32155800	0.66279400	-2.92371400
C	3.62631600	-0.81601200	-2.18495800
C	3.73547400	0.10603100	-3.21580600
C	2.61213400	1.03748500	-3.55095000
C	4.91221900	0.16233800	-3.95054600
H	4.99019800	0.87604900	-4.75653700
C	5.96995900	-0.67229400	-3.67057900
C	5.86147400	-1.59191800	-2.64314500
C	4.69630200	-1.65893600	-1.91461400
H	-0.86556300	-0.33438700	0.74068800
H	-0.90807700	-0.02105900	-1.76453600
H	2.75372400	0.60781600	0.06368500
H	1.09503000	-1.79545300	0.92683700
H	2.87134600	2.05337700	-3.24561700
H	6.87415700	-0.61198900	-4.25365100
H	6.67968900	-2.25603600	-2.41808800
H	4.60321200	-2.38211400	-1.11784800
H	1.35371900	-0.35270000	1.87588200
H	0.13879000	1.00111000	0.22317300
H	-0.29458800	-1.59403000	-1.29274300
H	2.13246000	-2.01122700	-1.29446200
H	2.47624700	1.09300100	-4.63192000
H	3.37239100	-0.95710500	0.54885200
H	0.43759700	1.17393400	-3.27698000
C	0.84503200	-1.61508700	-4.16968400
O	-0.34990200	-1.52348400	-4.40141500
H	1.57686700	-1.06183000	-4.74743200
H	1.17988000	-2.36963700	-3.46470300
F	-1.64279500	-0.91828900	-6.27327000
F	0.23743000	0.28982900	-5.81052400
F	-1.57075100	0.45387100	-4.44372100
B	-0.89800200	-0.31172100	-5.33774400

TS3-AE

E(RmPWB95) = -982.006663610 au

1 imaginary frequencies -204.1141 cm-1

C	0.00000000	0.00000000	0.00000000
C	0.00000000	0.00000000	1.42617700
C	1.25893000	0.00000000	2.07581400
H	1.44356000	1.08281100	1.68630600
O	1.23118200	2.15988900	0.33644800
C	0.20652000	1.59401300	-0.32001700
C	1.34677700	-0.12780800	3.57610400
C	-1.20924700	0.42469300	2.16723100
C	-1.14944900	-0.06082000	3.61377100
C	0.12354100	0.42702200	4.27379700
C	-2.50261900	0.18208800	1.43122000
C	-2.52059700	-0.21021100	0.10133300
C	-1.26585200	-0.47586900	-0.67046900
C	-3.74079400	-0.38600400	-0.53596300
H	-3.74678500	-0.69347600	-1.57041500
C	-4.92990300	-0.17148400	0.12073900
C	-4.91195300	0.23787600	1.44012500
C	-3.70749700	0.41340100	2.08191600
H	2.25033100	0.35964900	3.92718500
H	2.02813800	-0.51606300	1.51336700
H	-1.20239800	-1.14869700	3.63279400
H	0.14324200	1.51617000	4.24457100
H	-1.18051100	-1.54544300	-0.85358600
H	-5.86553200	-0.31215800	-0.39451500
H	-5.83329200	0.42538800	1.96596800
H	-3.70721400	0.74716100	3.10606400
H	0.13591200	0.14252500	5.32188800
H	1.45335200	-1.18463400	3.81576500
H	-1.06816200	1.51640700	2.21057200
H	-1.33861300	-0.01091500	-1.65218200
H	-2.01287700	0.30409600	4.15736000
H	0.89292300	-0.46042800	-0.40048300
H	0.36865900	1.57319600	-1.39683700
H	-0.74124100	2.07636300	-0.08153100
F	2.49248300	2.53386800	-1.59431600
F	2.82271000	0.61891500	-0.41893800
F	3.48238300	2.61224500	0.47952000
B	2.57743200	2.00243000	-0.33295400

22

E(RmPWB95) = -982.055751849 au

C	0.15948600	0.16900100	0.19239200
C	0.30540800	0.44929400	-1.26228700
C	1.28489700	-0.02220300	-2.02725700
C	2.34808900	-0.94639300	-1.49760800
C	2.45715000	-0.78299000	0.01329400
C	1.09347600	-0.93121600	0.65373200
C	1.37651300	0.34434400	-3.47506900
C	3.67435300	-0.79323800	-2.20512700
C	3.85815500	0.09264400	-3.25939900
C	2.73682600	0.95338600	-3.75619300
C	5.10081300	0.17130000	-3.87176100
H	5.23299800	0.85797300	-4.69397500
C	6.15474100	-0.61080700	-3.45882900

C	5.96909500	-1.50718900	-2.42355200
C	4.73882700	-1.59428700	-1.81217600
H	-0.87532900	-0.08203400	0.41704700
H	-0.41729700	1.11583000	-1.71463500
H	2.86802700	0.20305300	0.22859200
H	0.67817600	-1.90020100	0.37682500
H	2.77765800	1.92838400	-3.27378500
H	7.11111600	-0.53417200	-3.94929400
H	6.77781000	-2.14130100	-2.09936200
H	4.59574300	-2.30743400	-1.01544500
H	1.17496400	-0.92482100	1.73699200
H	0.36205200	1.09137000	0.73980200
H	2.01894000	-1.97525500	-1.68509700
H	2.85900900	1.13231900	-4.82279100
H	3.14970000	-1.51262900	0.42167200
H	0.59287100	1.05771600	-3.71292900
C	1.13691500	-0.88669800	-4.32393300
O	-0.11452600	-1.48839500	-3.95193700
H	1.04577500	-0.64753900	-5.37601000
H	1.90962900	-1.63728100	-4.19064300
F	-1.19329900	-1.58016400	-6.03090200
F	-1.37498900	0.32498000	-4.81092900
F	-2.43195800	-1.56339200	-4.09566000
B	-1.42470500	-1.03664900	-4.82116700
H	-0.27454000	-1.31896300	-3.01502800

MC4
E(RmPWB95) = -982.023346052 au

O	-0.51003300	1.97008100	0.86745500
C	-0.67412700	1.26001300	1.83595100
C	0.24789000	-1.55676200	1.84476400
C	-0.53339800	-1.43891200	0.77594800
H	-0.73345600	-0.17010100	-0.88403600
C	-0.02956600	-0.92943400	-0.53301300
C	-1.93741800	-1.94092900	0.73180300
H	-2.60161700	-1.12988900	0.43401300
C	-2.03583900	-3.06328300	-0.29564600
C	-0.10161700	-2.07519500	-1.54837800
C	-1.51639900	-2.61133000	-1.64673600
C	1.32953700	-0.30077100	-0.45299800
C	2.13772100	-0.42694000	0.66641900
C	1.69545800	-1.21748500	1.85914200
C	3.37967800	0.19265900	0.68230300
H	4.00296300	0.09353200	1.55855800
C	3.82319500	0.92720200	-0.39324600
C	3.01432800	1.05979900	-1.50740800
C	1.78004700	0.45343000	-1.52770500
H	-3.06627900	-3.40034800	-0.37730700
H	-2.25076300	-2.29065700	1.71264400
H	0.58187000	-2.86491300	-1.23632500
H	-2.16562300	-1.82588100	-2.03484200
H	2.27002100	-2.14497200	1.91781800
H	4.78962300	1.40307100	-0.36165400
H	3.34158400	1.64562100	-2.35049200
H	1.13631600	0.57619700	-2.38427300
H	-1.55673200	-3.43291000	-2.35810100
H	-1.44801000	-3.91227700	0.05318500
H	1.95104900	-0.67718400	2.77247200
H	0.24172700	-1.71981900	-2.51691900

H	-0.16515100	-1.98910100	2.74695700
H	-1.64148500	0.81104900	2.04147100
H	0.15415900	1.12009800	2.52332900
F	-2.13855400	3.51608900	0.16260300
F	-2.65190700	1.29826900	0.09307200
F	-1.15260100	2.15745900	-1.38824300
B	-1.74986200	2.28182200	-0.19004200

TS41-AE

E(RmPWB95) = -982.008796859 au

1 imaginary frequencies -222.4454 cm-1

O	0.00000000	0.00000000	0.00000000
C	0.00000000	0.00000000	1.28134600
C	1.76352200	0.00000000	1.99071000
C	2.09049600	-1.28521300	1.61664000
H	1.91519700	-1.21318200	-0.40061300
C	2.73023000	-1.47602900	0.29671400
C	1.67003000	-2.43194300	2.46350100
H	0.60465800	-2.56673800	2.26764800
C	2.35943300	-3.74320000	2.14521000
C	3.18551800	-2.89870900	-0.01295900
C	2.35182100	-3.97071200	0.65006400
C	3.81834500	-0.43965000	0.07755600
C	3.66491200	0.83171800	0.60569000
C	2.38835300	1.18123700	1.29512100
C	4.65930900	1.77689500	0.43590100
H	4.53143400	2.75948000	0.86326000
C	5.79884800	1.47874800	-0.28071500
C	5.94100400	0.22430900	-0.83589900
C	4.95909700	-0.72837800	-0.65254400
H	1.84885400	-4.54350300	2.67252400
H	1.76278000	-2.15536700	3.51113200
H	4.21325800	-3.01238600	0.33069200
H	1.33335500	-3.94631400	0.27366300
H	2.54898200	1.98180800	2.01086900
H	6.56701400	2.22313700	-0.41014700
H	6.81885100	-0.01905600	-1.41135500
H	5.08747300	-1.70014300	-1.09633000
H	2.77006700	-4.94506000	0.41276000
H	3.38620600	-3.72689600	2.50950200
H	1.70268200	1.56257700	0.53772000
H	3.19533600	-3.02829300	-1.09090900
H	1.49258200	0.12994100	3.02973500
H	-0.46393400	-0.83560600	1.79224000
H	-0.17416000	0.97158600	1.73275500
F	-1.80767400	-1.11148600	-0.97047900
F	-0.22237000	-2.32195700	0.12790300
F	0.28367900	-1.34826500	-1.86786600
B	-0.47861500	-1.25102100	-0.73910200

IN4

E(RmPWB95) = -982.010362860 au

O	-1.63134000	-1.81210100	0.11779600
C	-1.51245700	-1.62142200	-1.19485600
C	-0.09281900	-0.87343900	-1.57640700
C	-0.19682100	0.44447200	-1.04014000
H	-0.23855200	-0.07146900	0.88589200

C	0.34309900	0.66954500	0.29748600
C	-0.91005800	1.47689200	-1.80397400
H	-1.93872200	1.10499100	-1.83688900
C	-0.89109500	2.87104600	-1.21179100
C	0.14318400	2.06457800	0.87403900
C	-1.03790100	2.81110300	0.29305600
C	1.78761600	0.17495100	0.32987900
C	2.10521700	-0.98675600	-0.35461800
C	0.99757000	-1.75432200	-0.98743800
C	3.40928400	-1.44412000	-0.36238100
H	3.64951900	-2.34512900	-0.90479200
C	4.39477900	-0.76794800	0.32689900
C	4.07111800	0.36868600	1.03670600
C	2.77327500	0.84191400	1.03410300
H	-1.68716700	3.45368100	-1.66470600
H	-0.55793700	1.44664600	-2.83459200
H	1.03388500	2.65378800	0.66567200
H	-1.96779900	2.32389100	0.56094000
H	1.37407000	-2.42274300	-1.75537300
H	5.40848400	-1.13282300	0.31730100
H	4.82706600	0.89485400	1.59574300
H	2.53823900	1.72381400	1.60322800
H	-1.05736700	3.81619200	0.70471200
H	0.04565400	3.36511200	-1.46732300
H	0.54172300	-2.37003800	-0.21279800
H	0.05876300	1.98107200	1.95301400
H	-0.10862900	-0.85149500	-2.66162200
H	-2.31538400	-1.04574500	-1.64849900
H	-1.37561100	-2.56511300	-1.72194200
F	-3.77226100	-1.46798400	1.00271500
F	-2.61105900	0.29434400	0.13977400
F	-1.92916700	-0.65724000	2.09289900
B	-2.53241500	-0.92079500	0.88089000

TS42-AE

E(RmPWB95) = -982.008916112 au

1 imaginary frequencies -57.4643 cm-1

O	0.00000000	0.00000000	0.00000000
C	0.00000000	0.00000000	1.34091100
C	1.51872500	0.00000000	1.94190600
C	2.05192000	-1.22989300	1.43308200
H	1.62414300	-0.91041400	-0.35612500
C	2.61930100	-1.21094400	0.12238500
C	1.75097000	-2.48455400	2.14685400
H	0.66720600	-2.60068900	2.06473200
C	2.42775800	-3.71680200	1.58507900
C	3.06786600	-2.52571200	-0.48204200
C	2.31585700	-3.71159600	0.07634300
C	3.49848700	-0.01861200	-0.16271300
C	3.31693300	1.15703700	0.54244100
C	2.19095500	1.30179300	1.51276200
C	4.16312900	2.22647600	0.30443400
H	4.02640900	3.13876000	0.86450500
C	5.15984800	2.14493000	-0.64384200
C	5.31927000	0.98010100	-1.36557200
C	4.49372500	-0.09720500	-1.12147000
H	1.96405500	-4.59955500	2.01392600
H	1.95397200	-2.32764700	3.20554800
H	4.13484800	-2.64914700	-0.29833400

H	1.27116600	-3.65006500	-0.21525000
H	2.54255000	1.82586400	2.39772000
H	5.80416100	2.98965200	-0.82303600
H	6.08586000	0.90636300	-2.11886700
H	4.62508700	-1.00093400	-1.69015400
H	2.72316500	-4.62760600	-0.34100000
H	3.47666500	-3.73347700	1.87918700
H	1.43861900	1.93891200	1.05236300
H	2.92681600	-2.46409200	-1.55667900
H	1.36416600	-0.05921400	3.01413300
H	-0.53785400	-0.83273100	1.78251700
H	-0.37283600	0.94277200	1.74062500
F	-1.85159900	-0.92160700	-1.11265800
F	-0.54095000	-2.26961300	0.18270200
F	0.27673600	-1.45589200	-1.77151700
B	-0.58012100	-1.18165000	-0.71480600

23

E(RmPWB95) = -982.064134440 au

C	-4.08206100	-1.13844700	-0.00155800
C	-2.79199700	-1.75191400	-0.49739500
C	-1.66816900	-0.76709600	-0.56430800
C	-1.70760500	0.42899800	0.03600700
C	-2.87078400	0.83839700	0.89015300
C	-3.80107800	-0.30631000	1.22605400
C	-0.44252200	-1.21220200	-1.31686700
C	-0.58386600	1.37400400	-0.10235500
C	0.41429400	1.12775300	-1.04980500
C	0.26721800	-0.04180000	-1.97040700
C	1.49829700	1.97620700	-1.14570700
H	2.28176100	1.74024100	-1.84769300
C	1.60185200	3.08774100	-0.33462700
C	0.60793700	3.35606800	0.58219800
C	-0.46995000	2.50411700	0.69926900
H	-4.81310500	-1.91465400	0.20724900
H	-2.93319000	-2.18354100	-1.48803000
H	-3.41325500	1.64039200	0.38685000
H	-3.33527500	-0.93691600	1.98346900
H	-0.32651800	0.27637900	-2.82790400
H	2.46015100	3.73386600	-0.41399700
H	0.67680000	4.22164500	1.21996000
H	-1.22639100	2.73009500	1.42969800
H	-4.71919600	0.08051000	1.65969900
H	-4.50216800	-0.50231200	-0.78001400
H	-2.50731400	-2.58722800	0.14781500
H	1.24271600	-0.34732400	-2.33771000
H	-2.50080600	1.26749700	1.81747700
H	-0.75597500	-1.90446500	-2.09758600
C	0.46135400	-2.03142000	-0.41776400
O	1.01402900	-1.24453800	0.64710500
H	0.51895300	-0.43139700	0.79080400
H	1.31225900	-2.41676500	-0.96687400
H	-0.07485800	-2.85469800	0.04160900
F	3.12858400	-2.16003300	1.07608500
F	2.93164400	-0.69835700	-0.64148900
F	2.72880800	0.06058000	1.50419700
B	2.62504700	-0.98302300	0.65676300

MC5

E(RmPWB95) = -982.021858827 au

C	-0.06585800	-0.03561200	0.62791800
C	0.46453200	-0.22125300	-2.28318200
O	1.61868200	0.02148900	-2.55208900
C	2.65728900	-0.20649900	0.42107600
C	2.06371400	1.02984700	0.53912500
C	0.68976200	1.11780600	0.64653600
C	3.34739000	-5.46871100	0.83885800
C	2.40882100	-5.11764200	-0.30870400
C	1.70021800	-3.83862200	-0.01258200
C	2.61604200	-2.68487700	0.23773700
C	3.52690200	-3.03525900	1.41812300
C	4.28666000	-4.31824500	1.14441500
C	0.38658100	-3.75208900	0.11595100
C	1.90903200	-1.37582600	0.41493000
C	0.52929000	-1.28998300	0.52005200
C	-0.33535000	-2.51230600	0.50494100
H	-1.14160700	0.02535100	0.70945500
H	3.91179700	-6.36714100	0.60053200
H	1.69458000	-5.91747000	-0.48415200
H	2.91134000	-3.14569500	2.31067400
H	4.95040700	-4.16083700	0.29270200
H	-0.78277700	-2.64839900	1.49205000
H	0.21016100	2.07968900	0.71690800
H	2.66637100	1.92073300	0.50539700
H	3.72731900	-0.26891500	0.29694800
H	4.92161700	-4.56514700	1.99190100
H	2.75079400	-5.69158900	1.72312400
H	2.99771600	-5.00026300	-1.22067200
H	3.26811300	-2.58742900	-0.63596900
H	-1.18421000	-2.34935900	-0.16233200
H	4.21691600	-2.21611400	1.60654300
H	-0.21432800	-4.63785000	-0.03717900
H	-0.25954100	0.58194700	-2.17769700
H	0.16001300	-1.25858800	-2.17972500
F	3.28328800	1.66792100	-2.17213800
F	1.10849000	2.34313200	-2.31949500
F	2.20011300	1.61885100	-4.17803900
B	2.12048700	1.60141800	-2.83721200

TS5-DA

E(RmPWB95) = -981.985645664 au

1 imaginary frequencies -311.1416 cm-1

C	0.00000000	0.00000000	0.00000000
C	0.00000000	0.00000000	1.65465500
O	1.26677200	0.00000000	2.12401400
C	2.51045000	0.18811600	0.52206600
C	2.11821000	-1.05684100	-0.05131000
C	0.81863400	-1.15370500	-0.34582500
C	3.68894600	5.19405400	-1.04963300
C	2.50365200	5.18536000	-0.09632500
C	1.79401400	3.87362800	-0.15041500
C	2.67331200	2.69120700	0.11776600
C	3.86051400	2.70801300	-0.84707900
C	4.61079100	4.02244100	-0.77212500
C	0.51145200	3.76615600	-0.45645500
C	1.92313800	1.40432800	0.05999000

C	0.62206000	1.30435400	-0.24849600
C	-0.22945900	2.47939100	-0.54170500
H	-1.03393500	-0.06175600	-0.31515900
H	4.23016900	6.13299500	-0.96416800
H	1.81523300	5.99461600	-0.32277700
H	3.48421900	2.55202700	-1.85795900
H	5.04159200	4.13204600	0.22417500
H	-0.67798800	2.35794200	-1.53069100
H	0.35628900	-2.08590100	-0.62117300
H	2.79366400	-1.89274200	-0.04862800
H	3.44352400	0.22940000	1.06010900
H	5.44175600	4.01804700	-1.47292800
H	3.32076100	5.12980900	-2.07327000
H	2.87093000	5.34748800	0.91921600
H	3.07931300	2.79723000	1.12925000
H	-1.07862200	2.48119400	0.14845600
H	4.52441900	1.87487300	-0.62223000
H	-0.05506500	4.66398200	-0.65821300
H	-0.53985300	-0.89362100	1.94325400
H	-0.53560400	0.89885000	1.94453900
F	3.08168300	-1.28725400	2.75809300
F	1.09799400	-2.34207200	2.36139400
F	1.33081400	-1.04262500	4.21253200
B	1.71915800	-1.24492500	2.92603800

24

E(RmPWB95) = -982.005171061 au

C	-0.12399200	-0.08837300	-0.15146200
C	0.33300000	0.42293400	-1.52782400
O	1.75712000	0.28749900	-1.57971500
C	2.34428300	-0.20258700	-0.30956600
C	1.88833800	0.74807500	0.75085100
C	0.57308100	0.80500100	0.83428500
C	3.40913900	-5.31549900	1.15388200
C	2.39059500	-5.30805600	0.02281400
C	1.64541600	-4.01459500	-0.01571300
C	2.52673400	-2.80401300	-0.10008100
C	3.53712200	-2.82279400	1.04946900
C	4.32872400	-4.11453100	1.05886000
C	0.32511100	-3.94563500	0.05514800
C	1.72902200	-1.55354400	-0.12384600
C	0.41148500	-1.49069100	-0.05203300
C	-0.46424300	-2.68194500	0.03252800
H	-1.20473700	-0.04285400	-0.08555200
H	3.98374200	-6.23834900	1.13269100
H	1.69727800	-6.13931100	0.11665400
H	2.99219200	-2.71509900	1.98707900
H	4.91591500	-4.18100900	0.14172000
H	-1.09535500	-2.61393700	0.92229500
H	0.02590400	1.48197600	1.46742600
H	2.58202700	1.35436200	1.30425100
H	3.40736200	-0.20933100	-0.49553700
H	5.03708700	-4.11285400	1.88356300
H	2.88083100	-5.29032500	2.10673200
H	2.92184500	-5.43663000	-0.92298200
H	3.10262700	-2.87014400	-1.03021700
H	-1.16381800	-2.69133900	-0.80766600
H	4.20762800	-1.96876100	0.96858400
H	-0.23811200	-4.86531600	0.13084700

H	0.09812500	1.47197400	-1.66399600
H	-0.07472600	-0.16525100	-2.34050000
F	3.81859800	1.02831900	-2.38659200
F	2.37319400	2.57574000	-1.54867300
F	1.89724500	1.55784100	-3.52360600
B	2.54497800	1.48551500	-2.33939400
